# NF-κB-inducing kinase (NIK) is activated in pancreatic β-cells but does not contribute to the development of diabetes

**DOI:** 10.1038/s41419-022-04931-5

**Published:** 2022-05-19

**Authors:** Peng Xiao, Tatiana Takiishi, Natalia Moretti Violato, Giada Licata, Francesco Dotta, Guido Sebastiani, Lorella Marselli, Sumeet Pal Singh, Mozes Sze, Geert Van Loo, Emmanuel Dejardin, Esteban Nicolas Gurzov, Alessandra Kupper Cardozo

**Affiliations:** 1 Inflammation and Cell Death Signalling group, Laboratoire de Gastroentérologie Expérimental et Endotools, Université libre de Bruxelles, Brussels, Belgium; 2 Department of Medical Sciences, Surgery and Neurosciences, University of Siena, Siena, Italy; 3 Fondazione Umberto Di Mario, c/o Toscana Life Sciences, Siena, Italy; 4 Tuscany Centre for Precision Medicine (CReMeP), Siena, Italy; 5 Department of Clinical and Experimental Medicine, Islet Laboratory, University of Pisa, Pisa, Italy; 6 Institute for Interdisciplinary Research in Human and Molecular Biology, Medical Faculty, Université libre de Bruxelles, Brussels, Belgium; 7 Center for Inflammation Research, VIB, B-9052 Ghent, Belgium; 8 Department of Biomedical Molecular Biology, Ghent University, B-9052 Ghent, Belgium; 9 Laboratory of Molecular Immunology and Signal Transduction, GIGA-Insitute, ULiege, Liège, Belgium; 10 Signal Transduction and Metabolism Laboratory, Laboratoire de Gastroentérologie Expérimental et Endotools, Université libre de Bruxelles, Brussels, Belgium

**Keywords:** Diabetes, Stress signalling

## Abstract

The transcription factor nuclear factor-κB (NF-κB) has a key role in the pathogenesis of diabetes and its complications. Although activation of the canonical NF-κB pathway in β-cells is generally deleterious, little is known about the role of the non-canonical NF-κB signalling and its main regulator, the NF-κB-inducing kinase (NIK), on pancreatic β-cell survival and function. Previous studies based on models of NIK overexpression in pancreatic islet cells showed that NIK induced either spontaneous β-cell death due to islet inflammation or glucose intolerance during diet-induced obesity (DIO) in mice. Therefore, NIK has been proposed as a potential target for diabetes therapy. However, no clear studies showed whether inhibition of NIK improves diabetes development. Here we show that genetic silencing of NIK in pancreatic β-cells neither modifies diabetes incidence nor inflammatory responses in a mouse model of immune-mediated diabetes. Moreover, NIK silencing in DIO mice did not influence body weight gain, nor glucose metabolism. In vitro studies corroborated the in vivo findings in terms of β-cell survival, function, and downstream gene regulation. Taken together, our data suggest that NIK activation is dispensable for the development of diabetes.

## Introduction

Diabetes is one of the most prevalent chronic diseases worldwide, affecting more than 463 million people globally (9.3%). Projections show that if the rising trend of past decades continues, 700 million (10.9%) people will be diabetic by 2045 [1]. Type 2 diabetes (T2D), which represents 90% of diabetes cases, is characterized by a systemic chronic low-grade inflammation, insulin resistance and impaired function and survival of insulin-producing β-cells [2]. Type 1 diabetes (T1D), which accounts for around 10% of diabetic cases, is caused by autoimmune-mediated destruction of β-cells, leading to severe hyperglycaemia [3]. β-cell death is a feature of both T1D and T2D, highlighting the crucial need for a better understanding of this phenomenon and the development of interventions to preserve or restore β-cell mass.

Activation of NF-κB is key in the pathogenesis of diabetes and its complications [4]. In T1D, pro-inflammatory cytokines such as interleukin-1β (IL-1β), tumour necrosis factor (TNF) and CD40L, secreted by immune cells in the islets, induce the activation of NF-κB in β-cells via both the canonical and non-canonical pathways [5, 6]. Although, in vitro and in vivo models of T1D have shown that activation of the canonical NF-κB pathway in β-cells is generally deleterious [7], little is known regarding the role of the non-canonical NF-κB pathway in diabetes.

The non-canonical NF-κB pathway is characterized by the recruitment of cellular inhibitors of apoptosis 1 and 2 (cIAP1/2) by the TNF receptor-associated factor 2 (TRAF2) leading to TRAF3 proteolysis and accumulation of NIK. In turn, NIK phosphorylates IKKα leading to processing of inhibitory protein p100 into the active subunit p52 that binds to RelB and translocates to the nucleus to induce gene expression [8, 9]. Some ligands involved in T1D progression, such as lymphotoxin (LTα1β2), CD40L and the TNF superfamily 14 (TNFSF14, also named LIGHT) can activate the non-canonical NF-κB pathway [5, 8, 10, 11]. We have previously observed in vitro that knocking down of p100 decreased cytokine-mediated apoptosis and inflammatory responses in rodent β-cells [12], indicating a role for the non-canonical NF-κB pathway in β-cell demise. Recently, two studies showed that NIK overexpression in β-cells resulted in impaired glucose-stimulated insulin secretion (GSIS), however diverged regarding effects on islet inflammation and β-cell survival [13, 14]. While, transgenic overexpression of NIK specifically in β-cells (β-NIK-OE mice) resulted in early spontaneous diabetes onset in mice due to insulitis and β-cell death, mice expressing NIK constitutively in β-cells due to TRAF2/3 depletion showed no diabetic phenotype up to 16 weeks of age with a mild glucose intolerance under control chow diet [13, 14]. It is important to consider that sustained NIK overexpression is not representative of physiological conditions because NIK is constantly degraded [15, 16], and even in optimal stimulatory conditions its level is generally low. Thus, NIK overexpression is not optimal to study the effects of this kinase. To better understand the role of NIK and the non-canonical NF-κB in immune-mediated β-cell death we developed a β-cell-specific NIK knockout (*NIKβ*^KO^) mice. Under physiological conditions lack of NIK did not affect β-cell development or function and glucose homoeostasis. After multiple low-dose streptozotocin (MLDSTZ) treatment, metabolic parameters including glycemia, β-cell mass and recruitment of immune-cells to the islets were indistinguishable between *NIKβ*^KO^ and wild type (WT) littermates. Additionally, body weight gain and glucose metabolism were not different in *NIKβ*^KO^ mice as compared to their WT littermates after diet-induced obesity (DIO). Finally, we showed that specific ligands of the non-canonical NF-κB pathway did not affect β-cell death, cytokine/chemokine expression and insulin secretory function in mouse islets or human β-cells. Taken together, our data suggests that although NF-κB activation is involved in diabetes pathogenesis, the non-canonical NF-κB pathway led by NIK activation is dispensable for the development of diabetes in mice.

## Results

### NIK is not necessary for pancreatic β-cell development and the absence of NIK does not alter β-cell function and glucose homoeostasis in mice

**Fig. 1:**
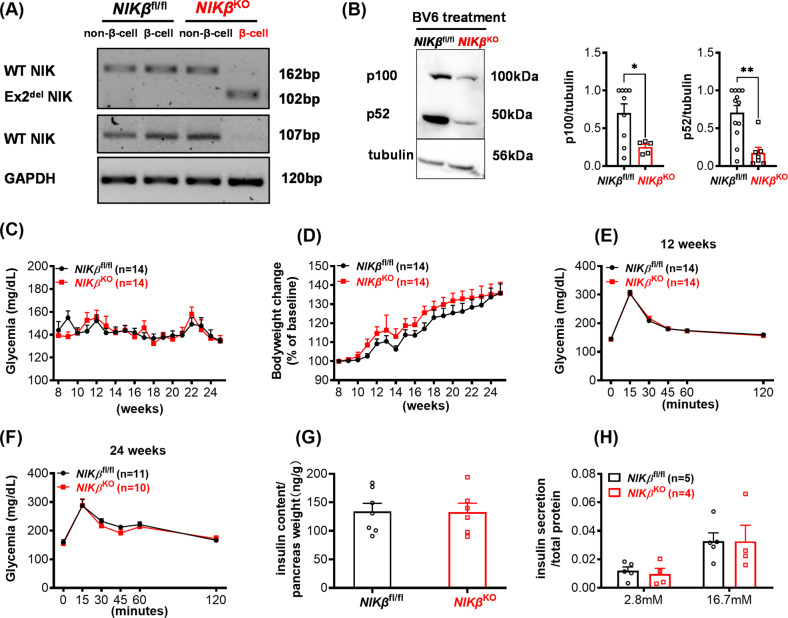
*NIKβ*^KO^ mice develop normally and have normal glucose metabolism. **A** RT-PCR for detecting WT and exon 2 deleted (Ex2^del^) NIK cDNA was performed on FACS purified pancreatic β- and non-β-cells. **B** Islets were treated as indicated for 24 h and p52, p100 levels were evaluated by western blot. Left panel, representative image. Right panel, quantitative analysis. *n* = 4–5. *p < 0.05, **p < 0.01. **C** Blood glucose and **D** bodyweight of male *NIKβ*^KO^ mice and WT littermates were followed weekly. IpGTTs were performed at **E** 12 and **F** 24 weeks and pancreatic insulin content was determined at 26 weeks (**G**). **H** GSIS of isolated islets. Means ± SEM. Unpaired *t*-test (**B** and **G**); mixed model ANOVA with post hoc Tukey test (**C**, **D** and **H**); 2-way ANOVA with post hoc Tukey test (**E,** **F**).

To assess the in vivo role of endogenous NIK expression in β cells, we generated *NIKβ*^KO^ by using NIK^fl/fl^ mice (gift from E. Dejardin) and crossing with RIP-CRE mice [17]. mRNA analysis of FACS purified β-cells from *NIKβ*^KO^ mice confirmed that they expressed a mutated NIK mRNA, while non-mutated NIK mRNA was detected in the non-β-cell populations from *NIKβ*^KO^ mice and both β-cell and non-β-cell populations from the WT mice (Fig. 1A). As shown in Fig. 1B, *NIKβ*^KO^ mouse islets had decreased expression of both p100 and p52 proteins as compared to WT mice, when treated with the second mitochondrial-derived activator of caspase (SMAC) mimetic BV6, which inhibits cIAPs leading to NIK stabilization [18]. This is expected since p100 is positively regulated by NIK [19, 20] and thereby we confirmed that *NIKβ*^KO^ mice had disrupted NIK signalling.

Male and female *NIKβ*^KO^ mice were monitored weekly for fed blood glucose and bodyweight and no difference was found between *NIKβ*^KO^ mice and WT littermates (Figs. 1C, D, S1A, B). At 12 and 24 weeks of age, NIK deletion did not affect glucose tolerance between genotypes (Figs. 1E, F, S1C). At 24 weeks of age, no differences in total pancreatic insulin content were detected between *NIKβ*^KO^ and WT mice (Fig. 1G). Finally, β-cell function was evaluated by performing GSIS and NIK depleted β-cells showed normal insulin secretory responses (Fig. 1H). Overall, our data suggest that *NIKβ*^KO^ mice have healthy β-cells and normal glucose homoeostasis under physiological conditions.

### NIK is dispensable for the development of immune-mediated diabetes in mice

**Fig. 2:**
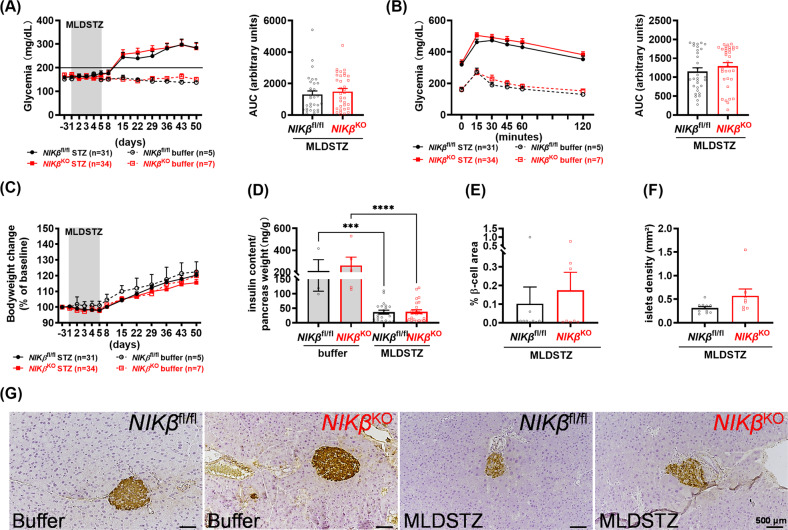
NIK absence in β-cells does not sensitize mice to MLDSTZ-induced T1D. Mice were treated with buffer or MLDSTZ as indicated. **A** Fed glucose levels (left panel). Right panel, area under the curve (AUC); and **C** bodyweight were determined. **B** IpGTTs were performed at 42 days after MLDSTZ (left panel). Right panel, AUC. **D** Total pancreatic insulin content was determined at endpoint of experiment. ***p < 0.001, ****p < 0.0001. MLDSTZ *n* = 21–25 mice; buffer treated *n* = 3–6 mice. **E** Beta-cell area and **F** mean islet density were evaluated. **G** Representative images of immunohistochemical staining for insulin; scale bars, 500 μm. MLDSTZ *n* = 8–10 mice; buffer treated *n* = 3–6 mice. Means ± SEM. Mixed model ANOVA with post hoc Tukey test (**A**, **B** left panels, **C**); unpaired *t*-test (**A**, **B** right panels, **E**, **F**); one-way ANOVA with post hoc Tukey test (**D**).

To verify if NIK activation played a role in immune-mediated β-cell death and diabetes development in vivo*, NIKβ*^KO^ and WT mice were administered MLDSTZ treatment. The low doses of STZ are specifically toxic to β-cells generating localized inflammation which is comparable to the inflammatory process described in human pancreas during T1D and the autoimmune nonobese diabetic (NOD) mouse model, namely, insulitis with initial attraction of neutrophils and macrophages followed by T cells, which causes progressive decrease in insulin levels due to β-cell destruction [21–24]. Both *NIKβ*^KO^ and WT mice developed hyperglycaemia at 7 days after last STZ injection (Fig. 2A). At the end of follow up, as expected, mice injected with MLDSTZ had impaired glucose tolerance as compared to buffer mice, although no difference between *NIKβ*^KO^ mice and WT littermates was observed (Fig. 2B). MLDSTZ treated *NIKβ*^KO^ and WT mice showed similar body weight during the experiment (Fig. 2C). The mice were sacrificed at 45 days after the last injection of STZ. At this time point, MLDSTZ-treated mice showed low levels of pancreatic insulin content, residual β-cell mass and islet density, while the difference was indistinguishable between genotypes (Fig. 2D–G). No difference was observed in the insulitis score between WT and *NIKβ*^KO^ mice (Fig. S2).

**Fig. 3:**
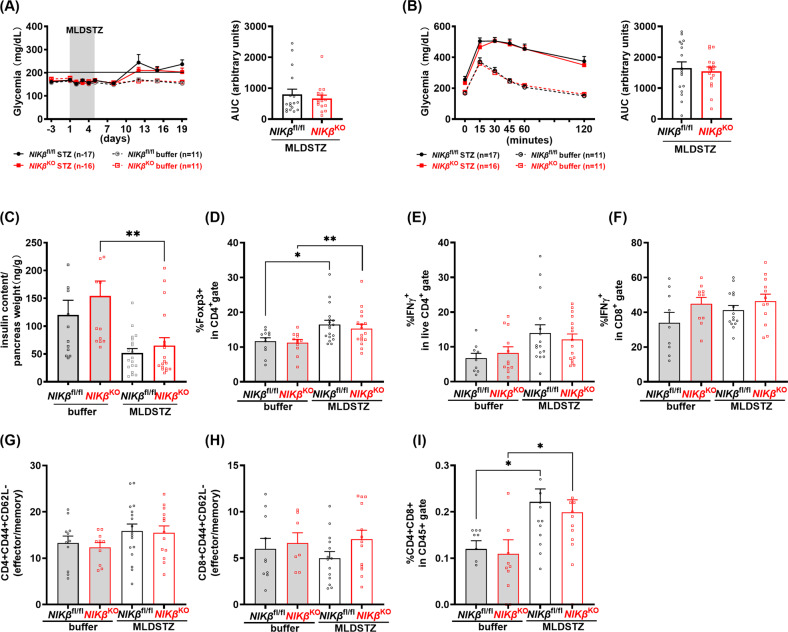
NIK absence in β cells does not modify MLDSTZ-induced insulitis. Mice were treated with buffer or MLDSTZ as indicated. **A** Fed glucose (left panel). Right panel, area under the curve (AUC). **B** IpGTTs were performed 12 days after MLDSTZ (left panel). Right panel, AUC. **C** Total pancreatic insulin content was determined at end of experiment. **p < 0.01. MLDSTZ *n* = 16–17 mice; buffer treated *n* = 11 mice. **D–I** 14 days after the MLDSTZ, pancreatic lymph nodes were harvested and FACS analysis was performed. Frequency of **D** CD4^+^Foxp3^+^ cells (Tregs), **E** CD4^+^ IFN-γ^+^ T cells (Th1), **F** CD8^+^ IFN-γ^+^ T cells, **G** and **H** effector/memory (CD44^+^CD62L^−^) CD4^+^ and CD8^+^. **I** Double positive CD4^+^CD8^+^ T cells are shown. *n* = 7–17 mice. Means ± SEM. *p < 0.05, **p < 0.01. Mixed model ANOVA analysis with post hoc Tukey test (**A**, **B** left panels); unpaired *t*-test (**A**, **B** right panels); one-way ANOVA with post hoc Tukey test (**C**–**I**).

A more refined analysis of immune responses was performed in MLDSTZ-treated mice sacrificed around 2 weeks after the last STZ injection, when full blow insulitis has been observed [23]. At this time point, mice are becoming hyperglycaemic and are glucose intolerant (Fig. 3A–C). However, no differences in these parameters were noticed between *NIKβ*^KO^ and WT mice.

We observed that MLDSTZ significantly increased the frequency of regulatory T cells (Tregs, CD4^+^Foxp3^+^) in pancreatic draining lymph nodes (pLN) compared to buffer controls, however no difference between *NIKβ*^KO^ and WT littermates were detected (Fig. 3D). In the spleen and blood, again increased frequency of Tregs were found in MLDSTZ mice but were similar between *NIKβ*^KO^ mice and WT littermates (Fig. S3A). Next, the frequencies of IFN-γ^+^Th1 (CD4^+^) and cytotoxic (CD8^+^) T cells were analysed, we found that Th1 were also increased in pLn of MLDSTZ-treated mice (although not statistically significance), cytotoxic T cells were not altered by MLDSTZ, and NIK absence did not affect frequency of IFN-γ^+^Th1 nor cytotoxic T cells (Fig. 3E, F). In the spleen, Th1 and cytotoxic T cells tended to be increased in MLDSTZ mice compared to controls, however no differences in blood or between *NIKβ*^KO^ and WT mice were found (Fig. S3B, C). We also analysed the frequencies of CD4^+^ and CD8^+^ effector/memory (CD44^+^CD62L^−^) T cells in pLn of mice and CD4^+^ effector memory T cells tended to be higher in MLDSTZ mice (although not statistically significant) (Fig. 3G, H). In blood, both CD4^+^ and CD8^+^ effector/memory cells were increased in MLDSTZ mice, no changes were found in spleen or between *NIKβ*^KO^ and WT mice (Fig. S3E, F). Interestingly, we found a significant increase of a rare population of double-positive CD4^+^CD8^+^ T cells linked to autoimmunity and chronic inflammatory diseases [25] in pLn of both *NIKβ*^KO^ and WT MLDSTZ-treated mice (Fig. 3I). Taken together, these results suggest that NIK absence in β-cell does not change in vivo glycaemic response nor inflammatory responses to islets in homoeostasis or MDLSTZ-induced diabetes.

### NIK in β-cells does not affect glucose tolerance nor insulin resistance in diet-induced obesity (DIO)

**Fig. 4:**
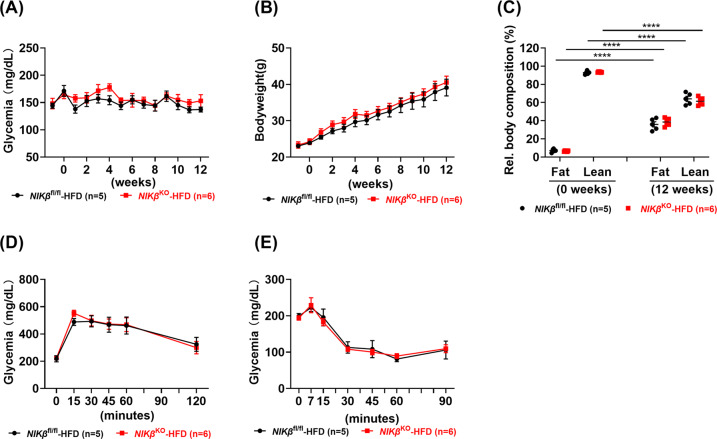
NIK depletion does not affect DIO. Mice were fed with a high-fat diet for 12 weeks. **A** Fed blood glucose and **B** bodyweight were determined. **C** Body mass were analysed. ****p < 0.0001. **D** IpGTTs and **E** iTTs were performed at 12 and 13 weeks after HFD, respectively. Means ± SEM. Mixed model ANOVA with post hoc Tukey test.

Next, we assessed the role of NIK in β-cells in DIO. During the 12 weeks of DIO, *NIKβ*^KO^ and WT littermates presented normal blood glucose (Fig. 4A) and the two mouse strains gained weight similarly (Fig. 4B). The nuclear magnetic resonance (NMR) analysis showed that after 12 weeks of HFD both *NIKβ*^KO^ and WT littermates had a significant increase in their fat mass, while a reduction in lean mass was observed. However, they showed equivalent percentages of fat mass and lean mass before and after DIO (Fig. 4C). Following the challenge with glucose, HFD-treated mice were glucose intolerant, but no difference was found between the two genotypes (Fig. 4D). Moreover, no different responses were observed at the level of insulin tolerance tests between *NIKβ*^KO^ and WT mice (Fig. 4E). These results demonstrate that NIK expression in β-cells does not influence the adverse metabolic consequences of DIO.

### NIK absence does not modify β-cell death neither affects inflammatory gene expression

**Fig. 5:**
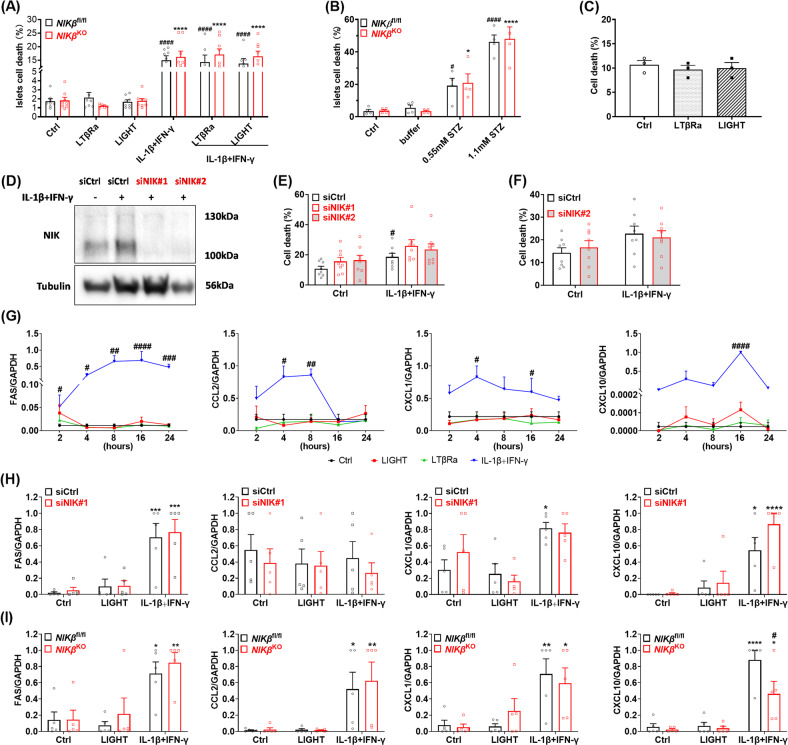
NIK absence does not change gene inflammatory expression pattern and neither modifies the sensitivity of mouse islets or human β-cells to streptozotocin (STZ) or cytokine-mediated β-cell death. Islets isolated were untreated or treated with **A** cytokines and/or the NIK ligands for 24 h or with **B** STZ for 30 min, as indicated, and the percentage of dead cells was determined. *n* = 4 to 8. ####*p* < 0.0001 vs. the *NIKβ*^fl/fl^ buffer, ****p < 0.0001 vs. the *NIKβ*^KO^ buffer. **C** EndoC-βH1 cells were untreated or treated with cytokines and/or the NIK ligands for 24 h, as indicated, and the percentage of dead cells was determined. *n* = 3. EndoC-βH1 cells (**D**, **E**) or dispersed human islets (**F**) were transfected with siRNAs against NIK (siNIK) and control (siCtrl) and treated with IL-1β + IFN-γ for 24 h. **D** Expression of NIK was assessed by western blot. A representative image is shown. *n* = 3. **E** and **F** The percentage of dead cells was determined. *n* = 3–8. #*p* < 0.05 vs. untreated siCtrl. **G** Gene expression analysis of EndoC-βH1 cells untreated or treated with cytokines and/or the NIK ligands, as indicated. *n* = 3. #*p* < 0.05, ##*p* < 0.01, ###*p* < 0.001, ####*p* < 0.0001 vs. the Ctrl condition. **H** Gene expression analysis of EndoC- βH1 transfected with siNIK or siCtrl, untreated or treated with cytokines for 16 h, as indicated. *p < 0.05, ***p < 0.001, ****p < 0.0001 vs. the respective untreated condition. *n* = 6. **I** Gene expression analysis of isolated islets untreated or treated with cytokines for 16 h, as indicated. *p < 0.05, **p < 0.01, ****p < 0.0001 vs. the respective Ctrl condition. #*p* < 0.05, vs. the respective *NIKβ*^fl/fl^ condition. *n* = 5. Means ± SEM. 2-way-ANOVA with post hoc Tukey test (**A**, **B**, **E**, **F**, **G**–**I**); one-way ANOVA with post hoc Tukey test (**C**).

We then further investigated the role of NIK on β-cell viability and inflammatory responses in islets from *NIKβ*^KO^ and WT mice and in the human β-cell line (EndoC-βH1 cells) [26, 27]. Treatment of mouse islets with ligands of the alternative NF-κB pathway, namely, lymphotoxin beta receptor agonist (LTβRa) [10, 28] or LIGHT did not induce islet cell death and showed no additive effect on the cell death mediated by IL-1β+IFN-γ. Moreover, islets from *NIKβ*^KO^ showed the same sensitivity to cell death as WT islets **(**Fig. 5A). Streptozotocin exposure led to the significant death of mouse islets cells in a dose-dependent manner, while no difference between *NIKβ*^KO^ and WT mouse islets was shown (Fig. 5B).

In the human β-cell line, the non-canonical NF-κB pathway was activated when treated with LIGHT, LTβRa (Fig. S4A–D). However, these specific ligands did not induce β-cell death (Fig. 5C). To verify the effect of NIK in human β-cell survival we knocked down NIK using siRNA (Figs. 5D, S4A, S4,C, S4,E, S4,G) and treated the cells with IL-1β+IFN-γ. IL-1β+IFN-γ-induced NIK stabilization and increased both the expression of p100 and p52 (Fig. S4G–H). However, NIK knockdown did not modify cytokine-mediated death of EndoC-βH1 cells or human islets (Fig. 5E, F).

We then compared how cytokines or NIK-specific ligands regulated NF-κB-dependent genes expression in human β-cells. Based on time course analysis, IL-1β+IFN-γ exposure significantly upregulated NF-κB-dependent genes Fas, Ccl2, Cxcl1, and Cxcl10 whereas LIGHT and LTβRa had no effect on the expression of these genes (Fig. 5G). To confirm that NIK did not affect Fas nor chemokine expression, we knocked down NIK in EndoC-βH1 cells exposed to IL-1β+IFN-γ or LIGHT for 16 h. As observed in Fig. 5H, expressions of Fas, Ccl2, Cxcl1, Cxcl10 were not modified by NIK silencing.

A similar effect was observed in mouse islets, in which neither LIGHT nor NIK absence had significant effect on gene expression, except for Cxcl10 that was decreased in islets from *NIKβ*^KO^ in response to exposure of IL-1β+IFN-γ (Fig. 5I). Overall, these data indicate that NIK absence has no major role in pancreatic β-cell death nor proinflammatory transcriptional regulation.

### Activation of non-canonical NF-κB does not affect insulin secretory function in mouse pancreatic islets and human β cells

**Fig. 6:**
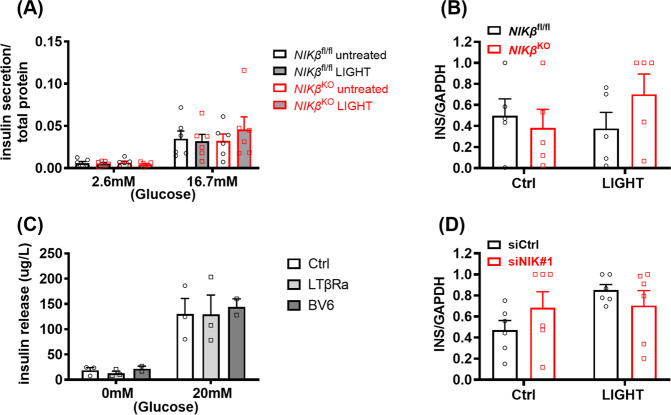
NIK activation does not affect function of mouse or human β-cells. **A** GSIS of islets untreated or exposed to LIGHT for 48 h. *n* = 6. **B** Gene expression analysis of isolated islets untreated (Ctrl) or treated with LIGHT for 16 h. *n* = 5. **C** GSIS of EndoC-βH1 cells were untreated or treated with LTβRa or BV6 for 24 h. *n* = 3. **D** Gene expression analysis of EndoC-βH1 transfected with siCtrl or siNIK, untreated or treated with LIGHT for 16 h. *n* = 6. Mixed model ANOVA analysis with post hoc Tukey test (**A** and **C**); 2-way-ANOVA with post hoc Tukey test (**B** and **D**).

NIK overexpression has previously been shown to inhibit GSIS in β-cells both in vitro and in vivo [13, 14]. Moreover, LIGHT has also been shown to inhibit GSIS in human islets [10]. However, in our study, exposure of WT or *NIKβ*^KO^ mouse islets to LIGHT had no effect on GSIS (Fig. 6A). The mRNA expression of insulin was also not regulated by LIGHT or NIK in mouse islets (Fig. 6B). In line with that, EndoC-βH1 cells did not show impaired insulin secretion when exposed to LTβRa and BV6 (Fig. 6C). Of note, BV6 induced NIK activation in EndoC-βH1 cells (Fig. S4E, F). Furthermore, exposure to LIGHT did not affect insulin mRNA expression in the presence or absence of NIK (Fig. 6D). These data go against a “physiological” effect of NIK on β-cell function.

## Discussion

NIK is the key kinase of non-canonical NF-κB signalling pathway, and its dysregulated expression has been found to play a role in many autoimmune diseases, such as systemic lupus erythematosus and rheumatoid arthritis [29, 30]. Contrary to the well-known involvement of the canonical NF-κB signalling in T1D and T2D islet inflammation [6, 31], the role of NIK and the non-canonical NF-κB signalling in diabetes pathology is unclear. Genome-wide association studies have identified a single-nucleotide polymorphism SNP rs17759555 of MAP3K14/NIK as a susceptibility gene of T1D [32] and recent studies using NIK overexpression in mice have shown negative effects of NIK on β-cell survival and function in models of diabetes. Importantly, NIK overexpression is not a physiological phenomenon and although NIK accumulation can temporarily occur under specific conditions to activate the non-canonical pathway, its expression is generally low due to constitutive ubiquitin-mediated protein degradation [8]. To overcome this issue, we developed a NIK floxed mice and produced a β-cell specific NIK KO mice (*NIKβ*^KO^), which enabled us to study the role of physiological NIK expression in diabetes development. Contrary to previous studies, *NIKβ*^KO^ mice did not show any abnormality in their glucose metabolism under physiological conditions, indicating that NIK is not necessary for embryonic development of β-cells and lack of NIK does not affect β-cell function [33].

To verify a possible role for NIK in immune-mediated β-cell death we exposed the mice to MLDSTZ to induce immune-mediated diabetes. We did not observe any differences in the incidence nor timing of diabetes development between *NIKβ*^KO^ and wild type mice. Moreover, the glycaemia levels of both *NIKβ*^KO^ and wild type littermates were similar. In agreement with these data, insulin content, β-cell mass and islet area were not affected by NIK absence in this model.

A previous study has shown that administration of a chemical inhibitor of NIK, B220, to high-dose STZ-treated mice improved the hyperglycaemia, glucose intolerance and even restored β-cell mass [13]. These results are surprising since we did not observe any protection in vitro when exposing *NIKβ*^KO^ mouse islets to different doses of STZ, neither did we observe a protection in mice treated with MLDSTZ. Of note, high dose STZ is not a model of inflammation mediated β-cell apoptosis, since it induces a fast and massive β-cell death mostly via necrosis [34, 35]. Another point to consider is that inhibitors often have non-specific targets. In line with this, a recent publication using a considered highly selective NIK inhibitor, named, SMI affects the activity of at least three other kinases, namely mitogen-activated protein kinase kinase kinase kinase 5 (MAP4K5), leucine-rich repeat kinase 2 (LRRK2), and protein kinase D1 (PKD1, PKCμ) [29, 36]. Thus, the use of chemical inhibitors of NIK which are less specific may lead to broader effects than observed by the outcome of specific genetic knockout models, such as our *NIKβ*^KO^.

In T1D, uncontrolled immune responses in the pancreas, particularly mediated by autoreactive T cells play a significant role in β-cell death [37]. Therefore, we compared different T cell subtypes in buffer and diabetic *NIKβ*^KO^ and *NIKβ*^fl/fl^ mice, at 2 weeks after last STZ injection, when T cells are described to be significantly increased in pLn of MLDSTZ-diabetic mice [38, 39]. In our study, MLDSTZ induced higher frequencies of CD4 effector T cells (IFN^+^, CD44^+^CD62L^−^), Tregs (CD4^+^Foxp3^+^) in pLn of diabetic mice, indicating as expected that MLDSTZ provoked significant inflammation in the pancreas. The accumulation of Tregs in inflamed sites, especially in the draining lymph nodes is conducive to optimal suppression of antigen-specific T effector responses [40, 41]. Moreover, a rare population of double positive (DP) CD4^+^CD8^+^T cells was also significantly increased in pLN of diabetic MLDSTZ mice, the DP T cells are present in healthy individuals but have been shown to be increased in several pathologies such as infections, neoplasias and some autoimmune diseases. They are described as having an effector/memory phenotype with enhanced cytolytic capacity [25, 42]. To our knowledge this is the first time that DP have been described in an autoimmune diabetic model. It turned out, however, that NIK deletion in β-cells did not elicit any differences in the T cell responses in either the local pLn or peripheral systems such as spleen and blood. Our results are significantly different from the results in β-NIK-OE mice, in which large infiltration of T cells correlated with extensive β-cell loss. This is probably due to the fact that forced NIK overexpression triggers both the canonical and non-canonical NF-κB activation [43–45].

Our in vitro data showed that NIK activation via specific ligands of the non-canonical NF-κB pathway did not induce death of human β-cells and islet cells. Moreover, inhibition of NIK was unable to prevent death of human β-cells and mouse islets induced by different conditions, such as IL-1β + IFN-γ or STZ. Additionally, we also observed in both human β-cells and mouse islets that the specific non-canonical ligands and NIK activation do not result in activation of Fas and several chemokines analysed indicating that the activation of these genes are occurring mostly via the canonical NF-κB pathway [13, 43, 46]. The only exception was Cxcl10, its expression seems to be modulated by NIK in mouse β-cells but not in the EndoC-βH1 cells. The differences observed in the two cellular models are probably due to species differences. It should also be considered that the mouse β-cells are primary non-dividing cells and the EndoC-βH1 is a cell line. Although expression of CXCL10 can be involved in insulitis [47–49], we did not observe any impact of the absence of NIK in our MLDSTZ model. This may indicate that absence of β-cell-mediated expression of CXCL10 is not enough to prevent insulitis or that in vivo CXCL10 expression was not modulated by NIK in β-cells. Overall, the in vitro data, reinforce the results obtained by the in vivo studies showing a disconnection between NIK activation and β-cell-mediated inflammation and β-cell death.

NIK overexpression due to TRAF2/3 depletion led to impaired insulin secretion in DIO mice, mostly via inhibition of GSIS glucose [10, 13, 14]. Our data in *NIKβ*^KO^ do not confirm any effects of NIK on glucose tolerance nor insulin resistance in DIO. Additionally, our present findings do not support a role for NIK in the regulation of β-cell function as we did not observe inhibition of GSIS neither modification of insulin mRNA expression in β-cells in conditions of NIK activation. Likewise, NIK absence/deletion in both mouse islets and human β-cells did not modify insulin mRNA expression. The different results between our studies and the previous one is that while in our study NIK is activated via its endogenous ligands, NIK overexpression can produce non-specific responses as discussed above.

Overall, our data suggests that ablation of NIK has no major effects in β-cells both in vitro and in vivo. Therefore, we postulate that NIK and the non-canonical NF-κB pathway do not play a significant role in β-cell insulitis and diabetes development.

## Materials and methods

### Materials

The cytokine concentrations utilized were based on prior studies [12, 50, 51] and are described in Supplementary Table 1. For NIK detection by western blots EndoC-βH1 cell were treated with the proteasome inhibitor MG-132 (Sigma-Aldrich, Diegem, Belgium) at 10 µmol/L for the last 8 h before being harvested.

### Culture and transfection of EndoC-βH1 and dispersed human islets cells

EndoC-βH1 cells were purchased from UNIVERCELL-BIOSOLUTIONS (MTA BH1-201601171) and cultured in low-glucose DMEM supplemented with 2% BSA fraction V, β-mercaptoethanol 50 µM, l-glutamine 1%, penicillin/streptomycin 2%, nicotinamide 10 mM, human transferrin 5.5 µg/mL and sodium selenite 6.7 ng/mL (all from Sigma Aldrich, Diegem, Belgium) [52]. The dispersed human islets from human organ donors were prepared as previously done [53]. Small interfering (si)RNAs (30 nmol/L) used are listed in Supplementary Table 2 and transfections were performed using lipofectamine RNAimax (Fisher Scientific, Aalst, Belgium) as described [50, 54, 55].

### Generation and characterisation of a β-cell-specific NIK knockout mouse strain, islet isolation, β-cell sorting and cell culture

NIK^fl/fl^ (gift from Prof. Dejardin, GIGA, University of Liege, Liege, Belgium) were crossed with RIP-Cre transgenic mice [56] to generate β-cell-specific NIK knockout mice *NIKβ*^KO^. Both lines are on the C57BL/6 genetic background and WT littermates were used as controls. *NIKβ*^KO^ mice were born at the expected normal Mendelian ratio.

The non-fasted-glycaemia and body weight were followed in male and female *NIKβ*^KO^ mice and their respective WT littermates from 8 to 24 weeks. An intra-peritoneal glucose tolerance test (ipGTT) was performed in these animals at 12 and 24 weeks of age. Mice were injected with 2 g/kg body weight glucose after 6 h of fasting. At 24 weeks, mice were sacrificed, and their pancreas collected for measuring the total pancreatic insulin content [57].

For islet isolation, mouse pancreases were digested by collagenase and incubated in a water bath at 37 °C. The islets were separated by a density gradient (Histopaque-1077; Sigma Aldrich), and then handpicked under a stereomicroscope [58]. These islets were cultured and treated as described [54]. For FACS purification, single mouse islet cell preparations were obtained, and the sorting of a β-cell-enriched cell populations was performed in a FACSAria instrument (BD Bioscience, San Jose, CA, USA) as described [59, 60]. RT-PCR using specific primers (Supplementary Table 4) designed for detecting exon 2 deletion of NIK were performed on FACS purified pancreatic β- and non-β-cells from both *NIKβ*^KO^ mice and WT littermates. Forward primer 1 is located at exon1 and forward primer 2 is at exon 2 which is flanked by loxP sites and deleted in *NIKβ*^KO^ mice, the common reverse primer is located at exon 3. GAPDH was used as loading control. Glucose-stimulated insulin secretion (GSIS) was performed in freshly isolated islets [57, 61]. Insulin was quantified using the Ultra-Sensitive Mouse Insulin ELISA Kit (Crystal Chem, Downers Grove, USA). The GSIS experiments were performed and measured in triplicates.

### Multiple low-dose streptozotocin treatment

Non-fasted male mice aged 7–8 weeks were randomly divided to be injected i.p. for 5 consecutive days with either 42.5 mg/kg body weight streptozotocin (Sigma-Aldrich, Belgium) dissolved in citrate buffer (100 mM pH ≤ 4.5, made freshly) or citrate buffer alone. Blood glucose levels were measured on days −3, 1, 2, 3, 4, 5 pre- and post-injection and later weekly during 7 weeks (for long term analysis) or 2 weeks (for short term analysis) after the last injection, in non-fasting conditions, using a glucometer (Accu-Chek, Roche, Switzerland) [62]. Hyperglycaemia was defined as non-fasting blood glucose levels >200 mg/dL in two sequential measurements. At the end of the experiment, the animals were sacrificed, and the pancreas collected for histological analysis or for measuring the insulin content.

### Diet-induced obesity model (DIO)

Male mice aged 8–9 weeks were randomly selected to be fed a high fat diet (HFD) containing 40 kcal% fat (mostly palm oil), 20 kcal% fructose and 2% cholesterol (D09100310i, Research Diets, New Brunswick, NJ) for 12 weeks. Glycemia and bodyweight were monitored weekly. Lean and fat mass were analysed using EchoMRI™ 3-in-1 (NMR) body composition analyzer (EchoMedical Systems, Houston, TX) at weeks 8 (prior to start of the diet) and 20 (12 weeks of the diet). Mice were fasted for 6 h for the ipGTT and 4 h for the insulin tolerance test (iTT).

### FACS analysis of immune cells

Single cell suspensions of pancreatic lymph nodes, spleen and blood were prepared from mice 14 days after last day of streptozotocin or buffer injections. The following antibodies were used for surface staining: CD45 (cat. 56-0451-82), CD3e (cat. 45-0031-82), CD8 (cat. 63-0081-80), CD44 (cat. 25-0441-81), CD62L (cat. 11-0621-81), CD11b (cat. 63-0112-80), Ly-6G/C (cat. 47-5971-80), F4/80 (cat. 45-4801-80), CD86 (cat. 25-0862-80), (all from eBioscience, San Diego, USA), CD4 (cat. A15384) (from Invitrogen, Merelbeke, Belgium) Intracelular mAb against IFN-γ (cat. 12-7311-81), FoxP3 (cat. 17-5773-82) were from eBioscience and used according to the manufacturer’s instructions. For viability staining, Zombie Violet™ Fixable Viability Kit (cat. 423113, Biolegend, San Diego, USA) was used. Data were acquired using a BD LSR Fortessa™ X-20 Cell Analyzer (BD) instrument running FACS DIVA software and were analysed using FlowJo v10 (TreeStar, Ashland, OR). Investigators analysing FACS data were blinded to mouse genotype and treatment groups.

### Histology of pancreas, beta cell fractional area and insulitis score analysis

Pancreata from sacrificed mice were collected and included in paraffin. To evaluate the beta-cell fractional area, formalin-fixed paraffin embedded (FFPE) tissue sections (5-μm thickness) were prepared by using a microtome (cat. RM2125 RTS-Leica Microsystems, Wetzlar, Germany) and baked overnight at 37 °C. After deparaffinization and rehydration through decreasing alcohol series (Xylene-I 20 min, Xylene-II 20 min, EtOH 100% 5 min, EtOH 95% 5 min, EtOH 80% 5 min, EtOH 75% 5 min) pancreatic tissue sections were incubated with 1× phosphate-buffered saline with Ca^2+^ and Mg^2+^ (PBS 1×) supplemented with 3% H_2_O_2_ (cat. H1009-Sigma Aldrich, St. Louis, MO, USA) for 40 min to block endogenous peroxidases. Heat-induced antigen retrieval was performed using 10 mM citrate buffer pH 6.0 in microwave (600 W) for 10 min, maintaining boiling conditions. Sections were incubated with PBS 1× supplemented with 3% bovine serum albumin (BSA, cat. A1470-25G, Sigma Aldrich, St. Louis, MO, USA) to reduce antibodies non-specific binding. Then, sections were incubated with primary antibody polyclonal Guinea Pig anti-Insulin (diluted 1:5 in 3% BSA, cat. IR002, Agilent Technologies, Santa Clara, CA, USA) for 1 h at RT. Subsequently, sections were incubated with secondary antibody Goat anti-Guinea Pig HRP-conjugate (cat. 106-036-003), Jackson ImmunoResearch, Philadelphia, PA, USA), diluted 1:2000 in PBS 1× for 1 h at room temperature (RT). Sections were then incubated with one drop of 3,3′-diaminobenzidine (DAB) chromogen solution (cat. RE7270-K, Novolink MAX DAB, Leica Microsystems, Wetzlar, Germany) for ~2 min to trigger the chromatic reaction. Stained sections were then counterstained with hematoxylin (cat. MHS31, Sigma Aldrich, St. Louis, MO, USA) for 4 min for better visualization of the tissue morphology. After the dehydration through increasing alcohol series, the pancreatic sections were mounted with Eukitt mounting medium (cat. S9-25-37, Bio Optica, Milan, Italy) and covered with a coverslip allowing them to dry. Images were acquired using optical microscope (cat. M570 E, Eclipse Ni-U, Nikon, Tokyo, Japan). For each section, islets were acquired at ×20 magnification and total section area were measured using NIS-elements viewer software analysis (vs. 4.40.00). For each islet, insulin positive area was evaluated using ImageJ software (vs. 1.8.0). The sum of each insulin positive islet area was normalized to the area of the whole section (reported as mm^2^) in order to obtain the β-cell fractional area. The islet density value was calculated by counting the total number of islets in the section and normalizing that with the area of the whole section (reported as mm^2^). Insulitis score was assessed as previously done [63], by assigning a score of the islet infiltration to each islet analysed, as follows: 0, no infiltration; 1, peri-insulitis; 2, islets with <50% of infiltration; 3, islets with >50% of infiltration. Investigators performing histological analysis were blinded to mouse genotype and treatment groups.

### Quantitative RT-PCR and Western blot analysis

Poly(A)^+^mRNA was isolated and reverse-transcribed as described [54]. The real-time PCR amplification reaction was performed using SYBR Green and compared with a standard curve [64]. Expression values were corrected for the housekeeping gene GAPDH. All primers used are listed in Supplementary Table 3.

For Western blot analysis, cells and islets were washed once with cold PBS and then lysed with RIPA buffer supplemented with proteinase cocktail inhibitor [65]. Denatured lysates were then resolved by SDS–PAGE and transferred to a nitrocellulose membrane. Western blot analysis was performed as described [65]. The following antibodies were utilized: anti-human NF-κB2 antibody (cat. 05-361, Merck KGaA, Darmstadt, Germany) [66]; anti-mouse NF-κB2 antibody (cat. 4882S, Cell Signaling technology, Leiden, The Netherlands), anti-NIK antibody (cat. 4994S, Cell Signaling technology, Leiden, The Netherlands) [12]; anti-GAPDH human polyclonal antibody (cat. 2275-PC-100, Trevigen, Gaithersburg, USA); polyclonal anti-tubulin (cat. T9026, Sigma-Aldrich Diegem, Belgium), and horseradish peroxidase-conjugated goat anti-rabbit (cat. P044801-2) or anti-mouse (cat. P044701-2) IgG from Agilent (Santa Clara, United States) [12, 58, 60].

### Assessment of cell viability

The percentages of viable cells were determined using the DNA-binding dyes propidium iodide (PI, 5 µg/mL, Sigma-Aldrich) and Hoechst 33342 (HO, 5 µg/mL, Sigma-Aldrich), as described [65]. For mouse islets, the percentages of dead cells were evaluated in a minimum of 10 islets per condition. All assessments were performed by two independent researchers one of whom was unaware of the identity of the samples.

### Statistical analysis

Data are presented as means ± SEM and were analysed using GraphPad Prism (version 9.3.1, GraphPad, USA). Shapiro–Wilk normality test was performed to confirm the normal distribution of the data using JASP (version 0.16.1, University of Amsterdam, Amsterdam, The Netherlands). The power and sample size were defined by the Web-based Sample Size/Power Calculator (provided by Dr. Rollin Brant, University of British Columbia, Canada) using the standard deviations calculated from at least three independent pilot experiments/cohorts of animals. A power of 80% and a significance of 5% were selected. The variances between compared groups were similar. Unpaired *t*-tests were used to compare the means of two independent groups. One-way ANOVA with Tukey’s multiple comparison were used to determine the differences between three of more independent groups. 2-way ANOVA tests with Tukey multiple comparisons were used to determine the differences of three or more groups with two independent variables. For tests between groups with repeated measurements mixed model ANOVA analysis for repeated measurement with Tukey’s multiple comparison was used. A *p*-value ≤ 0.05 was considered statistically significant.

## Supplementary information

Reproducibility checklist

Supplemental Data

Original Data File

## Data Availability

All data needed to evaluate the conclusions in the paper are present in the paper. Additional data related to this paper may be requested from the corresponding author.
