# Supplementary material for: NF-κB-inducing kinase (NIK) is activated in pancreatic β-cells but does not contribute to the development of diabetes
Source: Cell Death Dis. 2022 May 19;13(5):476. doi: 10.1038/s41419-022-04931-5 (PMC9120028; doi:10.1038/s41419-022-04931-5)
Supplement: Supplementary file 2 — Supplemental Data [file 41419_2022_4931_MOESM2_ESM.docx]

**Supplemental Data**

**NF-κB-inducing kinase (NIK) is activated in pancreatic β-cells but does not contribute to the development of diabetes**

P. Xiao^1*^, T. Takiishi^1*^, N. M. Violato^1^, G. Licata^2,3^, F. Dotta^2-4^, G. Sebastiani^2,3^, L. Marselli^5^, S. P. Singh^6^, M. Sze^7-8^, G. V. Loo^7-8^, E. Dejardin^9^, E. N. Gurzov^10^ and A. K. Cardozo^1^#


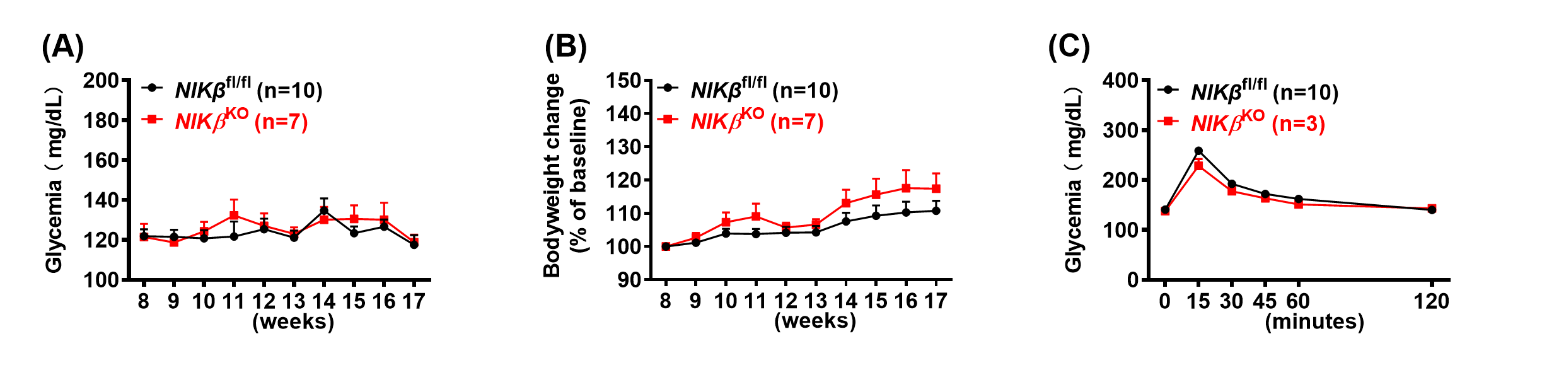


**Supplementary Figure 1. Female *NIKβ*^KO^ mice show normal glucose metabolism.** Female mice were followed weekly and (**A**) blood glucose and (**B**) bodyweight were determined. (**C**) IpGTT was performed at 12 weeks. Means ± SEM. Mixed model ANOVA with post hoc Tukey test.


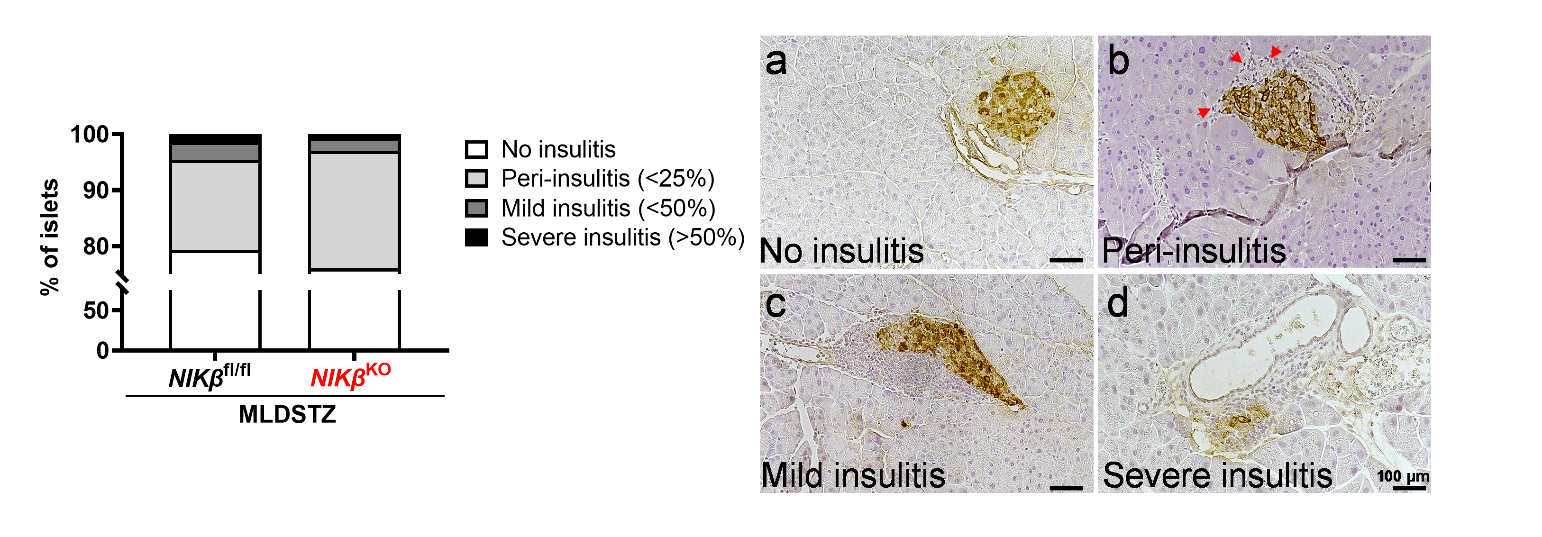


**Supplementary Figure 2. NIK absence in β-cells does not modify MLDSTZ-induced insulitis.** Insulitis score analysis of mouse pancreatic tissue sections was performed at 45 days after MLDSTZ. Insulitis was graded and a mean insulitis score **(left panel)** was calculated. Representative images of insulitis **(right panel)** are shown: (a) No insulitis; (b) peri-insulitis (red arrows indicating peri-islets immune cells); (c) Mild-insulitis; (d) severe insulitis; scale bar is 100 µm. n=8 -10 mice. Means ± SEM. 2way-ANOVA analysis with post hoc Tukey test.


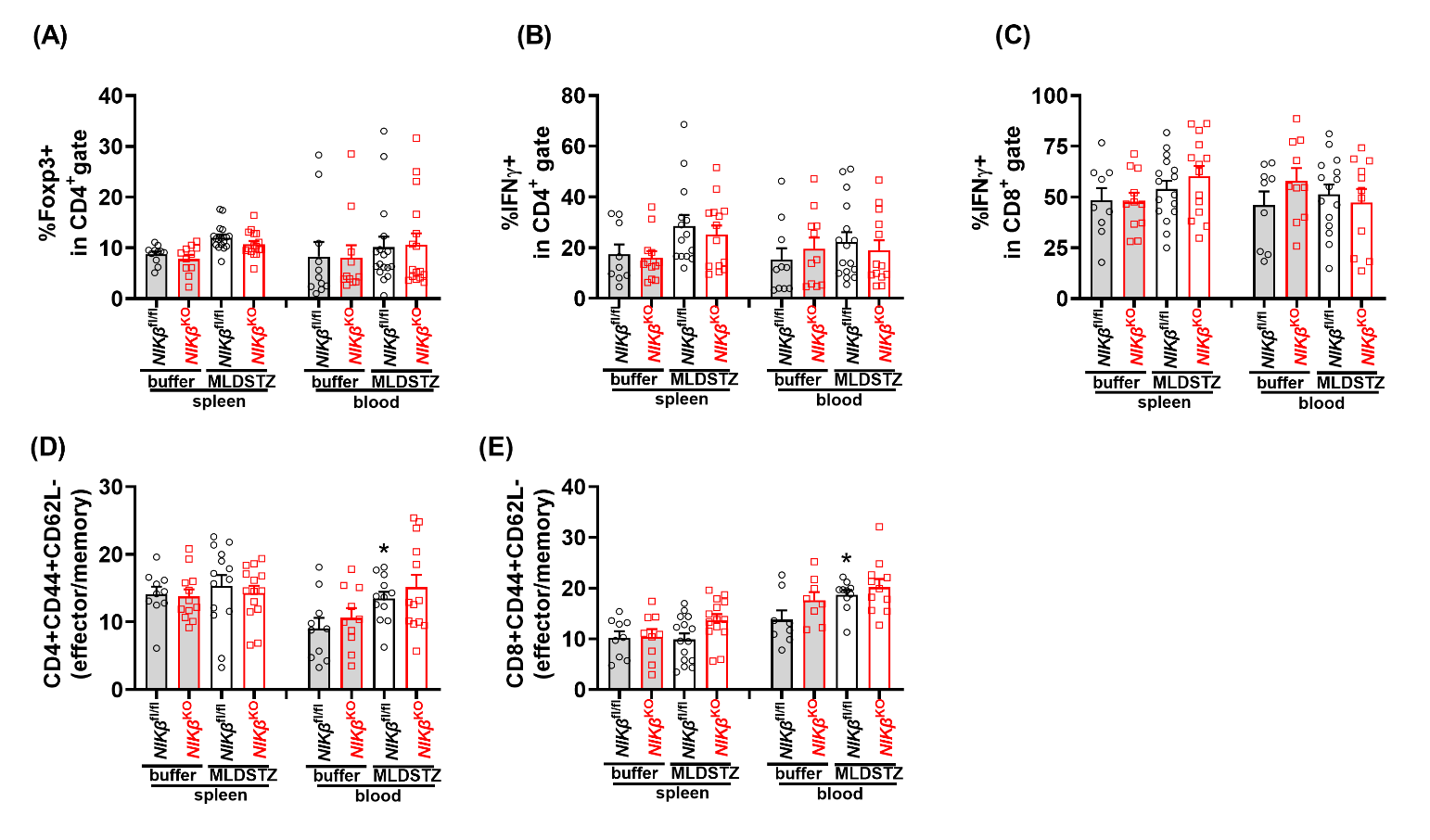


**Supplementary Figure 3. Immune T cell phenotype of *NIKβ*^KO^ mice do not differ from wild type littermates in systemic immune regulation during MLDSTZ mediated diabetes.** 14 days after MLDSTZ spleen and blood were harvested and FACS analysis was performed. Frequency of (**A**) CD4^+^Foxp3^+^, (**B**) CD4^+^IFN-γ^+^, (**C**)CD8^+^IFN-γ^+^ (**D-E**) effector/memory (CD44^+^CD62L^-^) CD4^+^ and CD8^+^ are shown. *p<0.05, **p<0.01 vs respective buffer treated animals. MLDSTZ n=6; Buffer treated n=8-10. Means ± SEM. One-way ANOVA analysis with post hoc Tukey test.


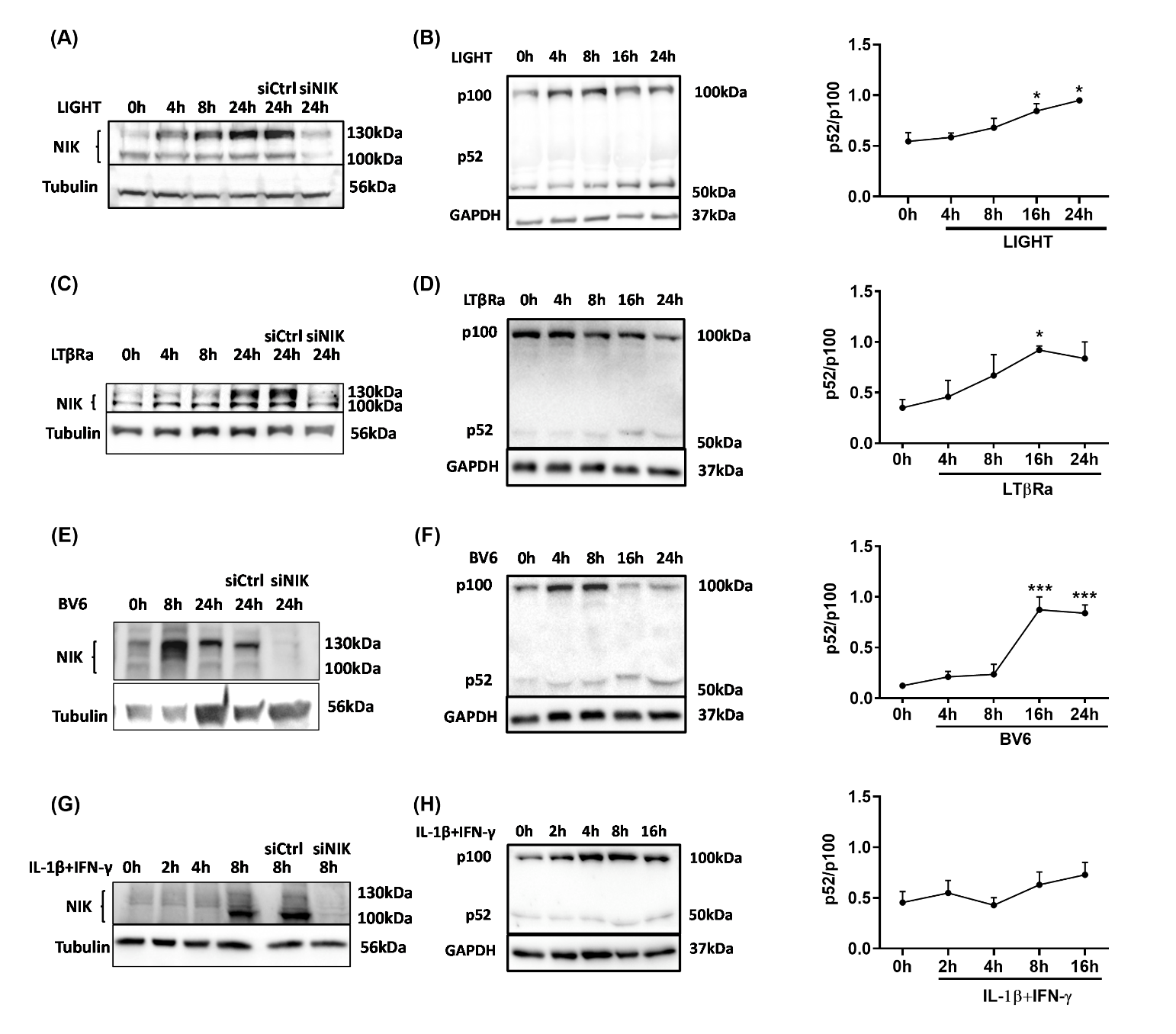


**Supplementary Figure 4. Specific ligands of the alternative NF-κB pathway and SMAC mimetic BV6 induce NIK stabilization and p100 to p52 conversion in human β-cells**. (**A-F**) EndoC-βH1 cells were left untreated (Ctrl) or treated with cytokines and/or the NIK ligands as indicated. **(A-C-E-G)** Expression of NIK was assessed by western blot (WB) in the presence of MG132 (10uM). As a control for the specificity of NIK bands, in some wells EndoC-βH1 cells were transfected with NIK (siNIK) or control (siCtrl) small interfering RNAs before treatment. Representative images. n=3-4. **(B-D-F-H)** Left panels, expression of p100, p52 and were assessed by western blot. Right panels, quantitative analysis. n= 3-6. Means ± SEM. One-way ANOVA with post Tukey test.

**Table 1. List of reagents used in human β-cells and mouse islets treatment.**

| \| **Reagent** \| **Species** \| **Company** \| **Concentration** \| \| --- \| --- \| --- \| --- \| \| IL-1β \| human \| R&D Systems, Abingdon, UK \| 50 U/mL \| \| IFN-γ \| human \| R&D Systems, Abingdon, UK \| 1,000 U/mL \| \| IFN-γ \| mouse \| R&D Systems, Abingdon, UK \| 1,000 U/mL \| \| TNF \| mouse \| R&D Systems, Abingdon, UK \| 1,000 U/mL \| \| MG-132 \|  \| Sigma-Aldrich, Diegem, Belgium \| 10 μmol/L \| \| LIGHT \| human \| R&D Systems, Abingdon, UK \| 100 ng/mL \| \| LIGHT \| mouse \| R&D Systems, Abingdon, UK \| 100 ng/mL \| \| SMAC mimetic BV-6 \|  \| Selleck Chemicals, Munich, Germany \| 5 μmol/L \| \| lymphotoxin beta R (LTβR) \| human \| R&D Systems, Abingdon, UK \| 200 ng/mL \| \| lymphotoxin beta R (LTβR) \| mouse \| R&D Systems, Abingdon, UK \| 200 ng/mL \|   **Table S2. List of siRNAs used for RNA interference.** | |
| --- | --- | --- | --- | --- | --- | --- | --- | --- | --- | --- | --- | --- | --- | --- | --- | --- | --- | --- | --- | --- | --- | --- | --- | --- | --- | --- | --- | --- | --- | --- | --- | --- | --- | --- | --- | --- | --- | --- | --- | --- | --- | --- | --- | --- | --- |
|  | |
|  |  |
| **siRNA** | **Sequence** |
| **human siRNA NIK#1** | GGAUUGACCUCACCCAGAA |
| **human siRNA NIK#2** | GAACCGGGCACUACAGCAA |
| **human siRNA Ctrl** | Qiagen |

**Table S3. List of primers used in qPCR analysis.**

**Human**

| **Gene** | **Forward** | **Reverse** |
| --- | --- | --- |
| GAPDH | ACAACTTTGGTATCGTGGAAGG | GCCATCACGCCACAGTTTC |
| FAS | TCTGGTTCTTACGTCTGTTGC | CTGTGCAGTCCCTAGCTTTCC |
| CCL2 | AGCCACCTTCATTCCCCAA | GGGTCAGCACAGATCTCCTT |
| CXCL1 | TTTTGAAATGTCAACCCCAAG | GATCTCATTGGCCATTTGC |
| CXCL10 | CCACGTGTTGAGATCATTGC | GCCTTCGATTCTGGATTCAG |
| INS | GCAGCCTTTGTGAACCAACAC | CCCCGCACACTAGGTAGAGA |

**Mouse**

| **Gene** | **Forward** | **Reverse** |
| --- | --- | --- |
| GAPDH | GCCTGGAGAAACCTGCCAAGTATGA | AACCTGGTCCTCAGTGTAGCCC |
| FAS | GCGGGTTCGTGAAACTGATAA | GCAAAATGGGCCTCCTTGATA |
| CCL2 | CCCAATGAGTAGGCTGGAGA | AAAATGGATCCACACCTTGC |
| CXCL1 | GCTTGAAGGTGTTGCCCTCAG | AAGCCTCGCGACCATTCTTG |
| CXCL10 | GCCGTCATTTTCTGCCTCAT | GCTTCCCTATGGCCCTCATT |
| INS | GGAAGCCCCGGGGACCTTCAGA | GGCGGGTCGAGGTGGGCCTTA |

**Table S4. List of primers used in RT-PCR analysis.**

| **Gene** | **Forward** | **Common reverse** |
| --- | --- | --- |
| **NIK** | Primer 1: CACAGCCAAGGACGGCTC | TGGGGATTTGATCGAGACTCT |
|  | Primer 2: GGCTGAATGTGAGAATAGCCAA |  |
