# Supplementary material for: NF-κB-inducing kinase (NIK) is activated in pancreatic β-cells but does not contribute to the development of diabetes
Source: Cell Death Dis. 2022 May 19;13(5):476. doi: 10.1038/s41419-022-04931-5 (PMC9120028; doi:10.1038/s41419-022-04931-5)

Figure 1B

p100/p52


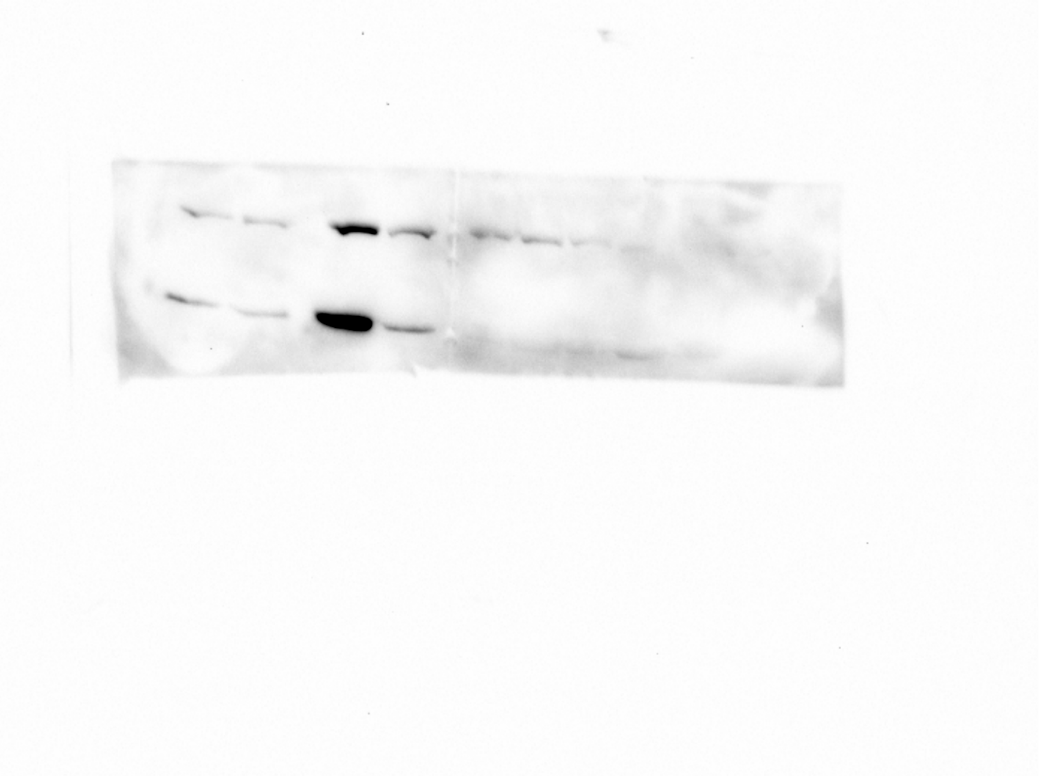


Tubulin


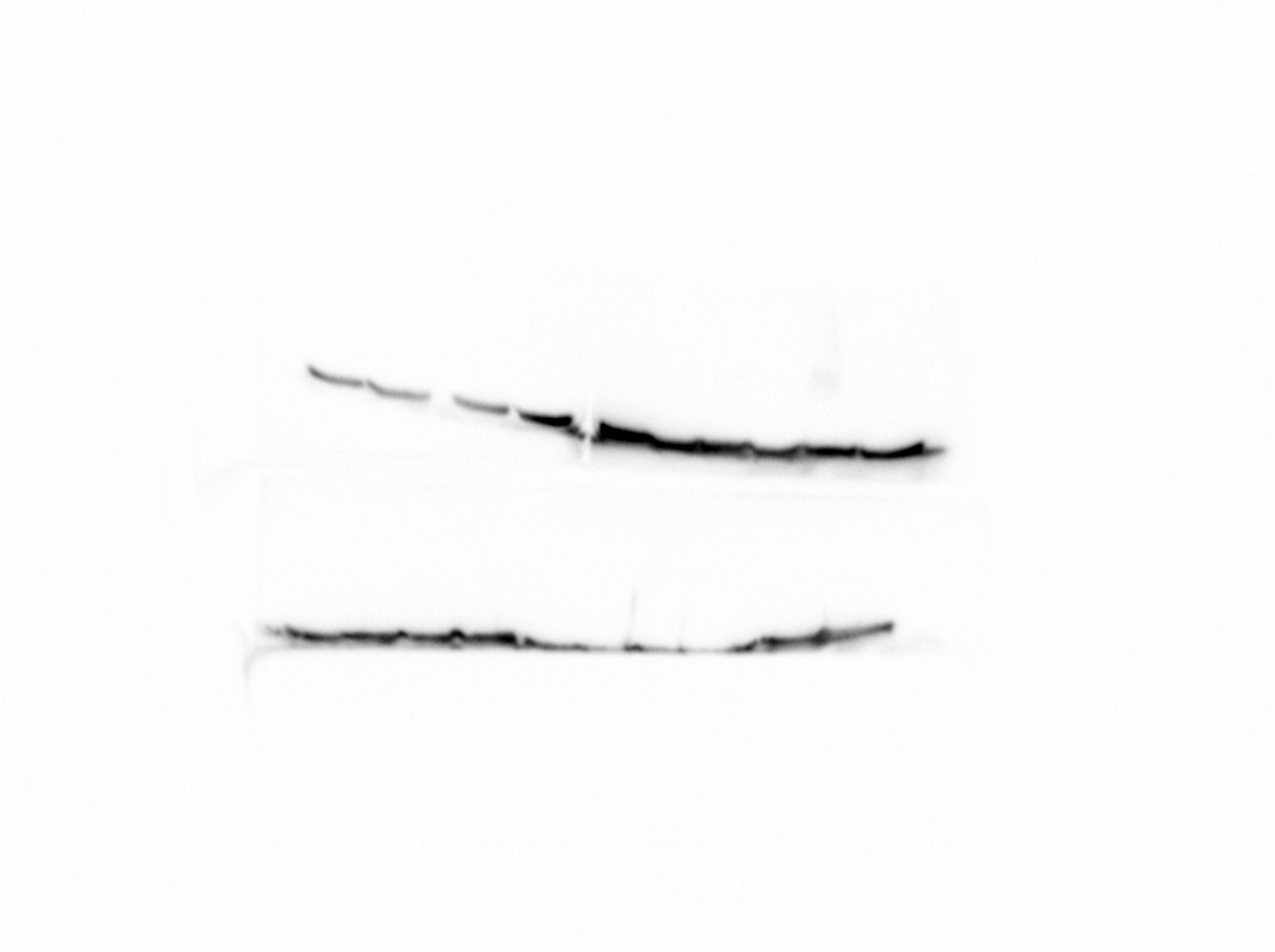


Figure 5D

NIK


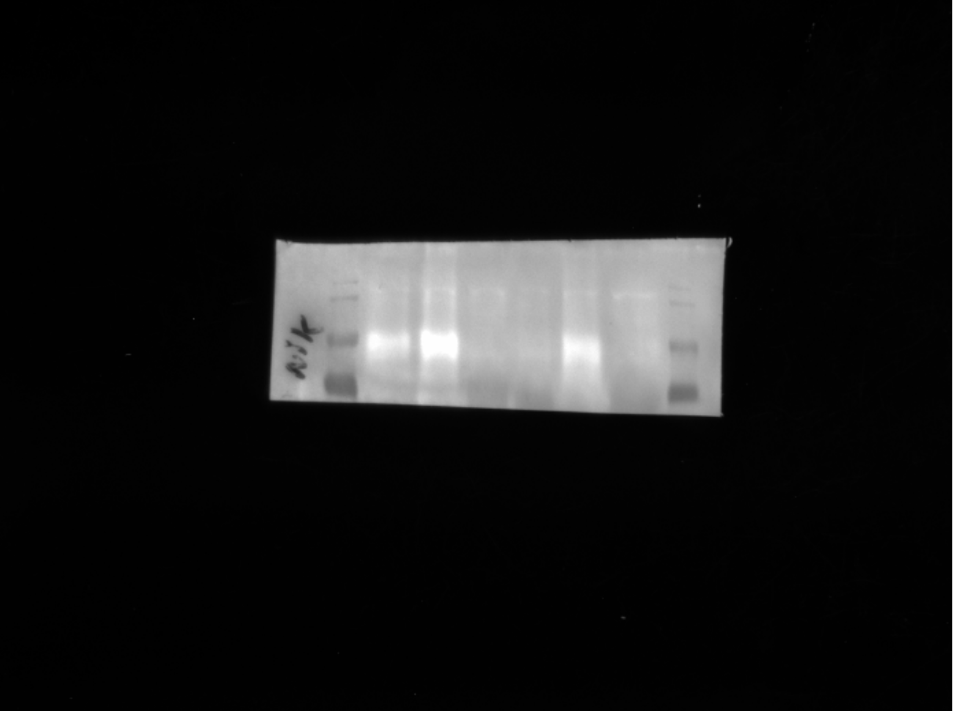


Tubulin


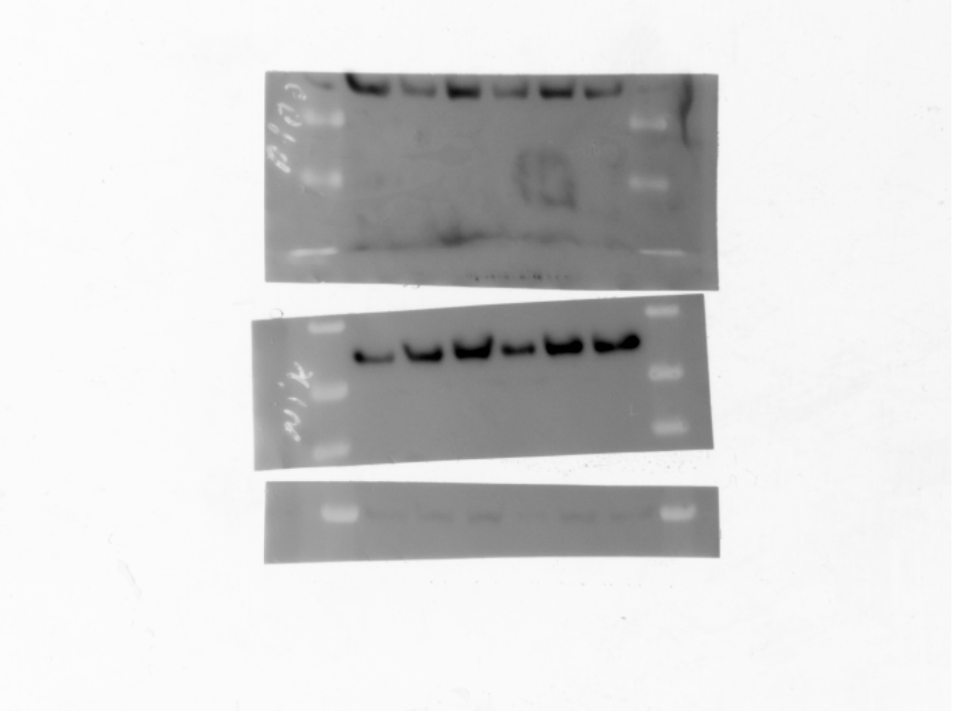


Figure supplementary 4A

NIK


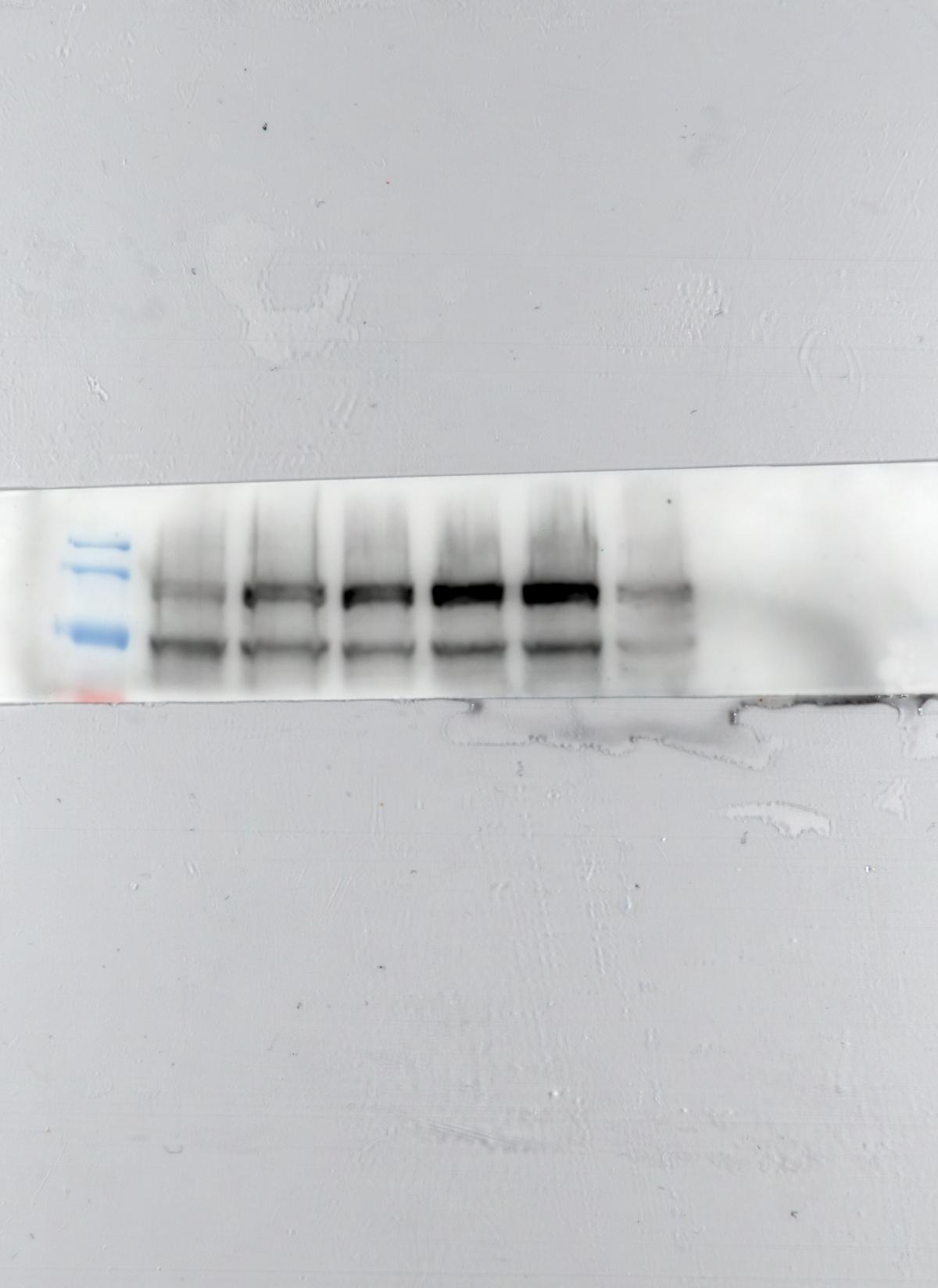


Tubulin


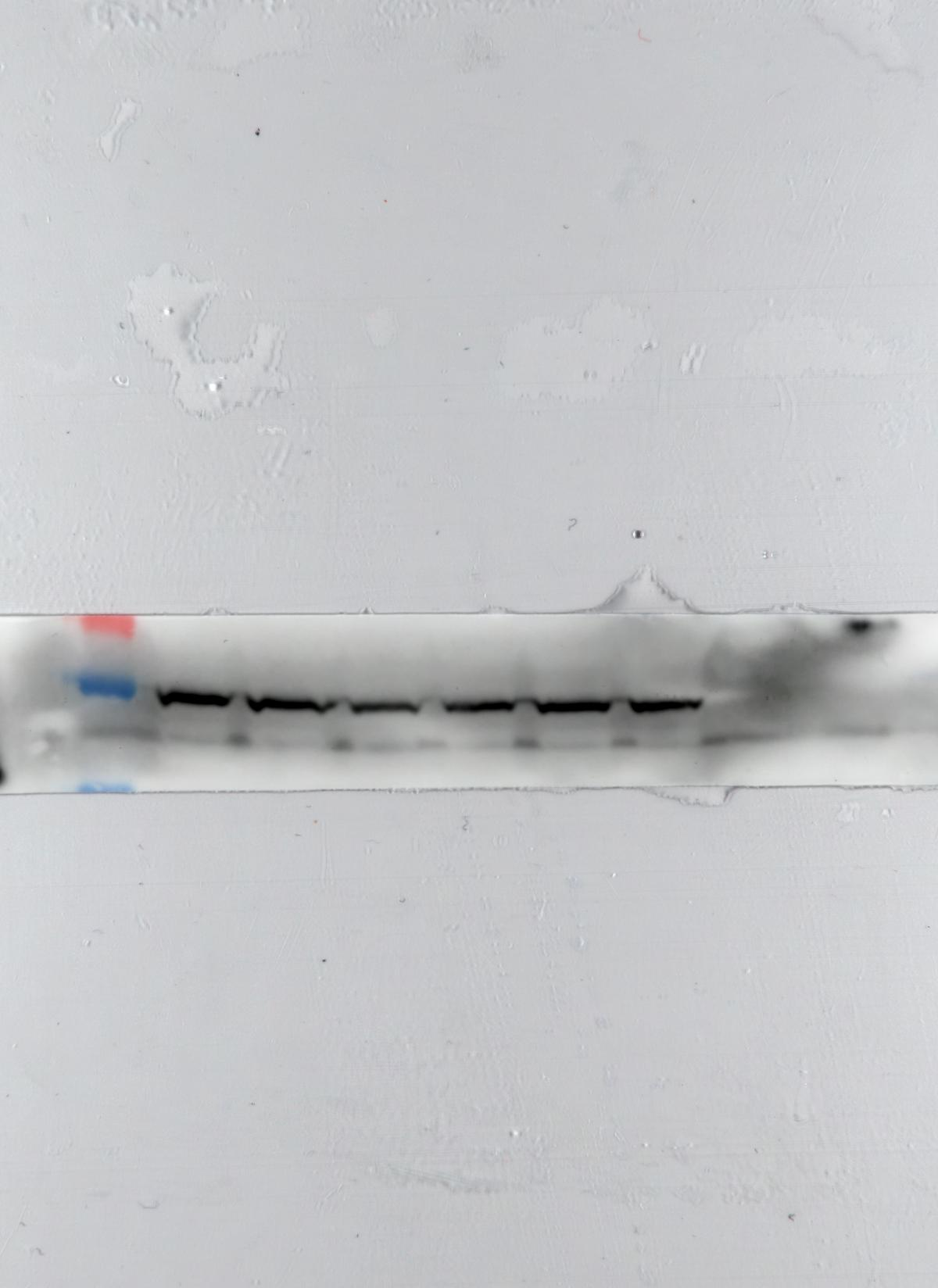


Figure supplementary 4B

P100/p52


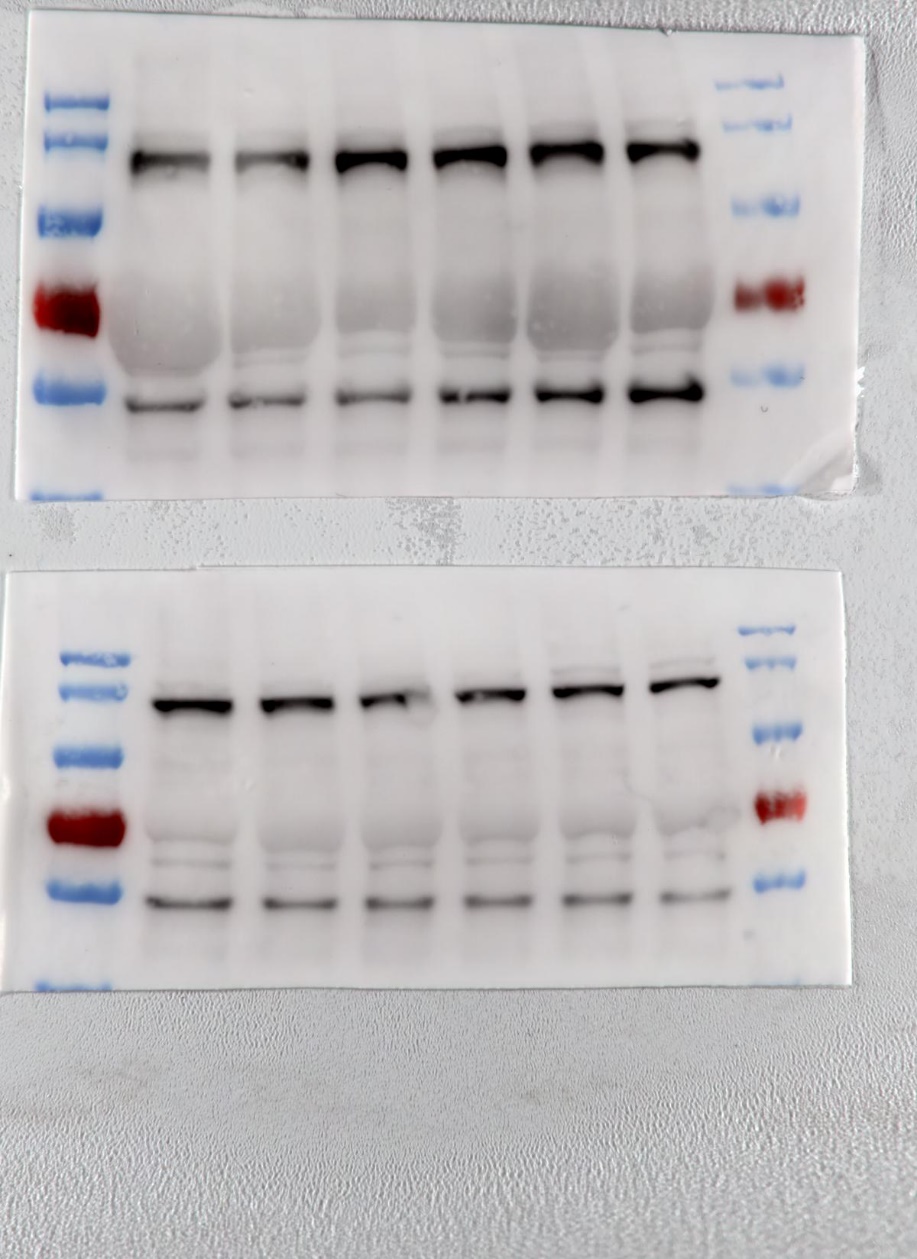


GAPDH


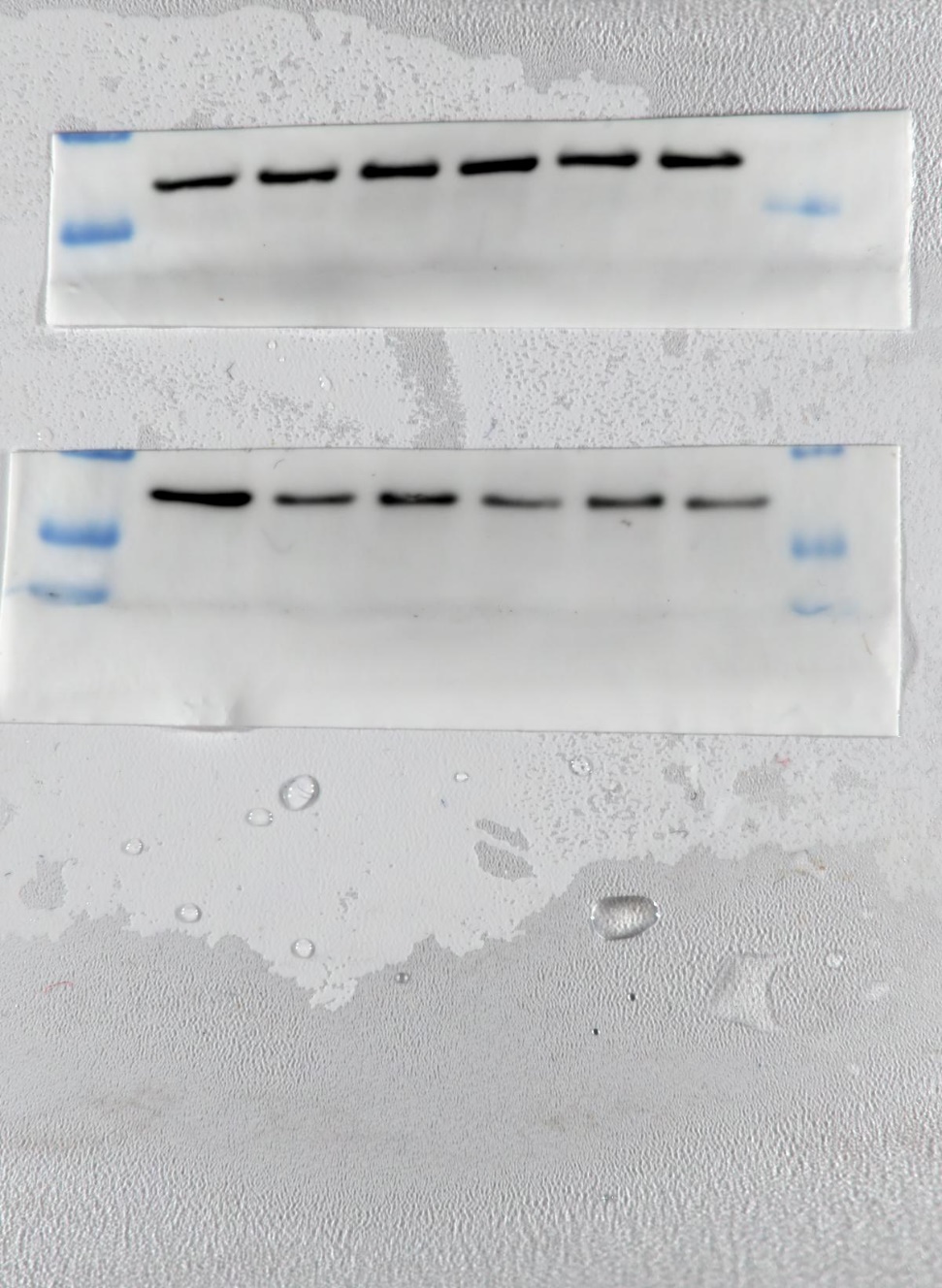


Figure supplementary 4C

NIK


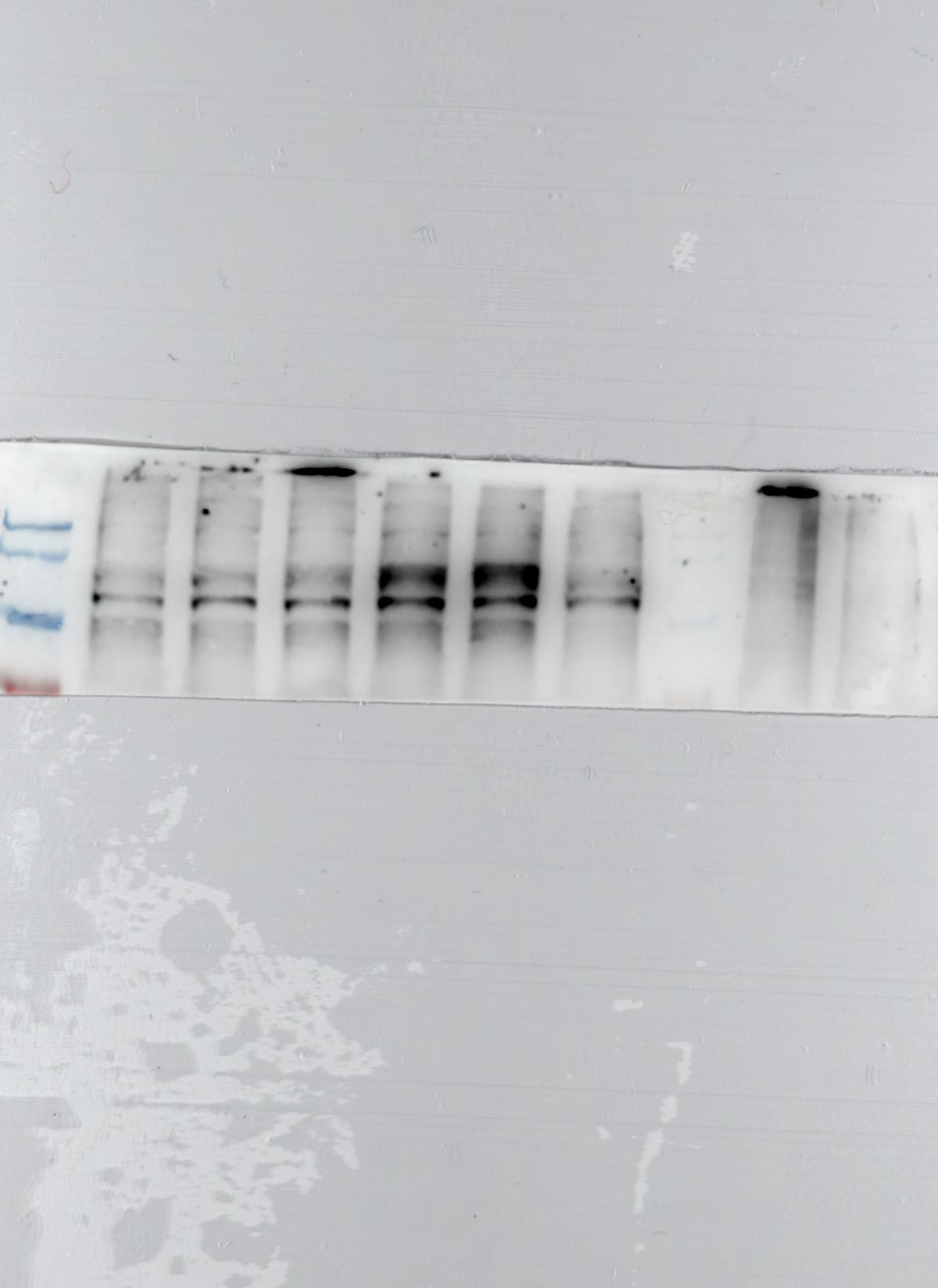


Tubulin


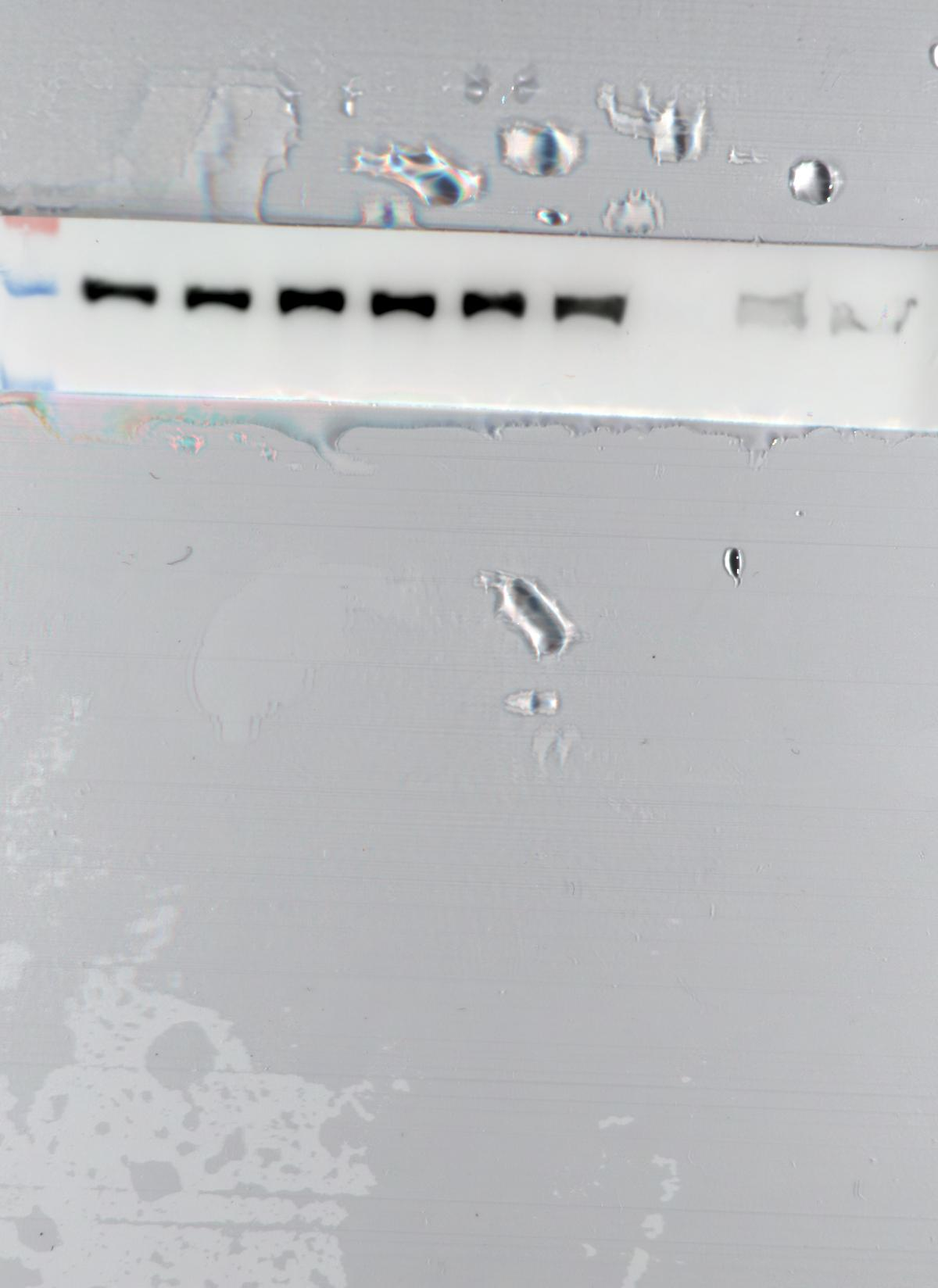


Figure supplementary 4D

P100/p52


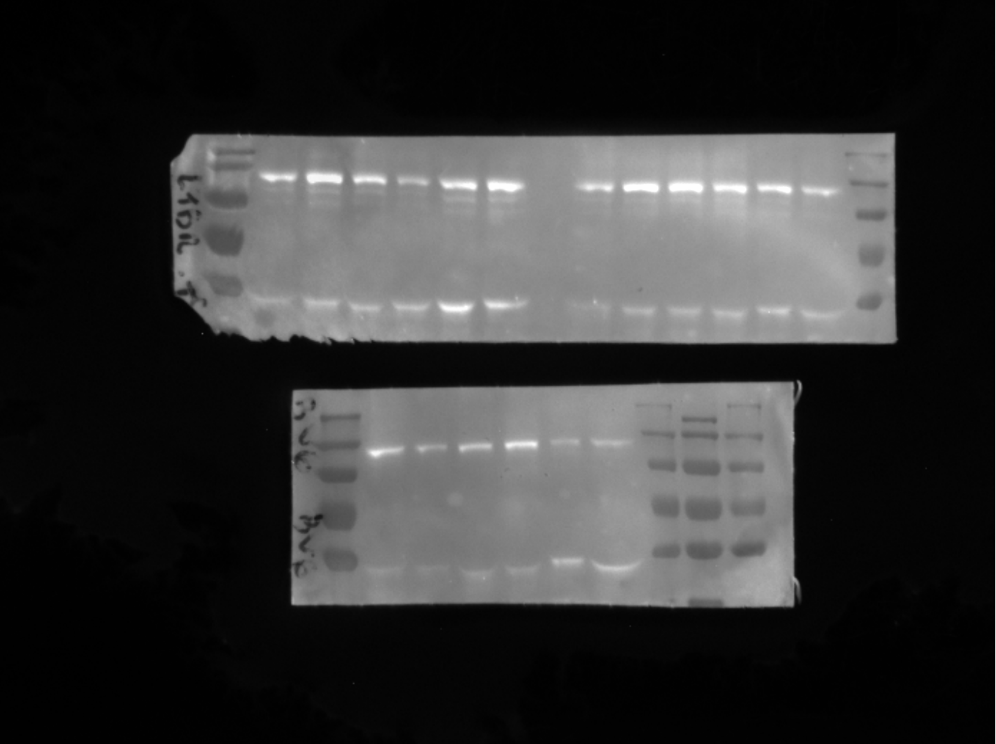


GAPDH


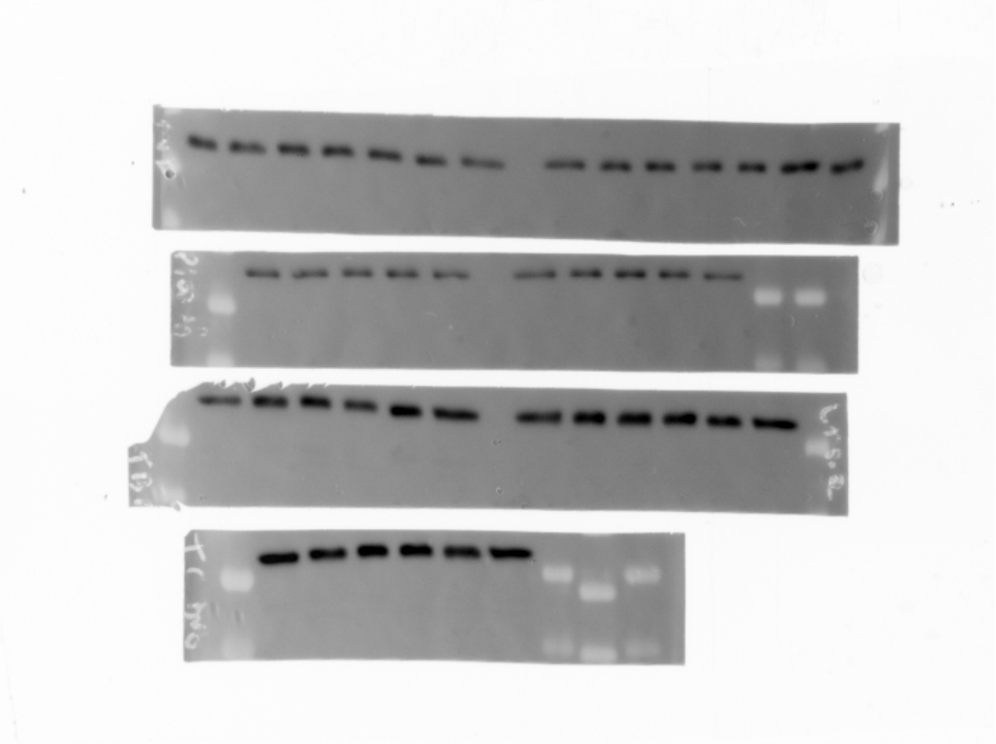


Figure supplementary 4E

NIK


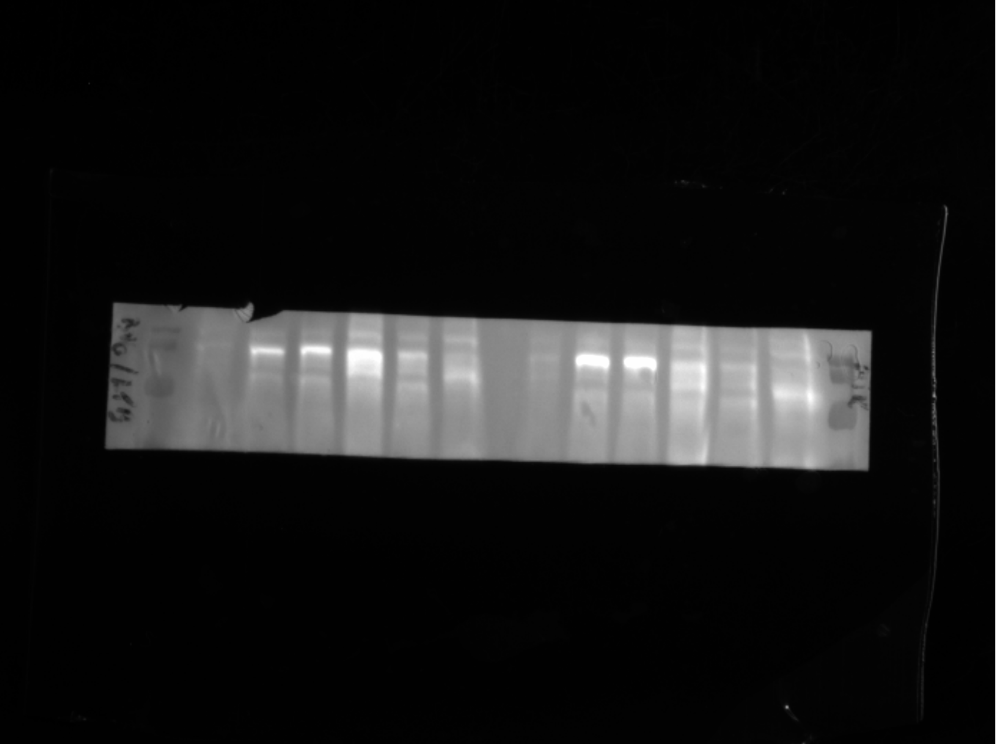


Tubulin


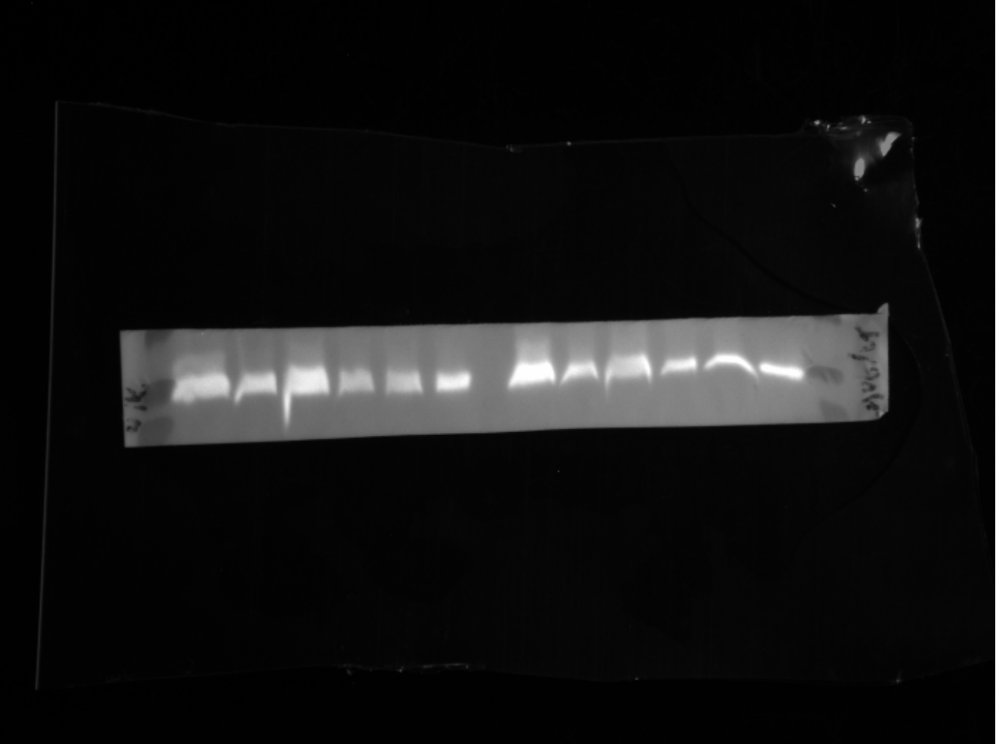


Figure supplementary 4F

P100/p52


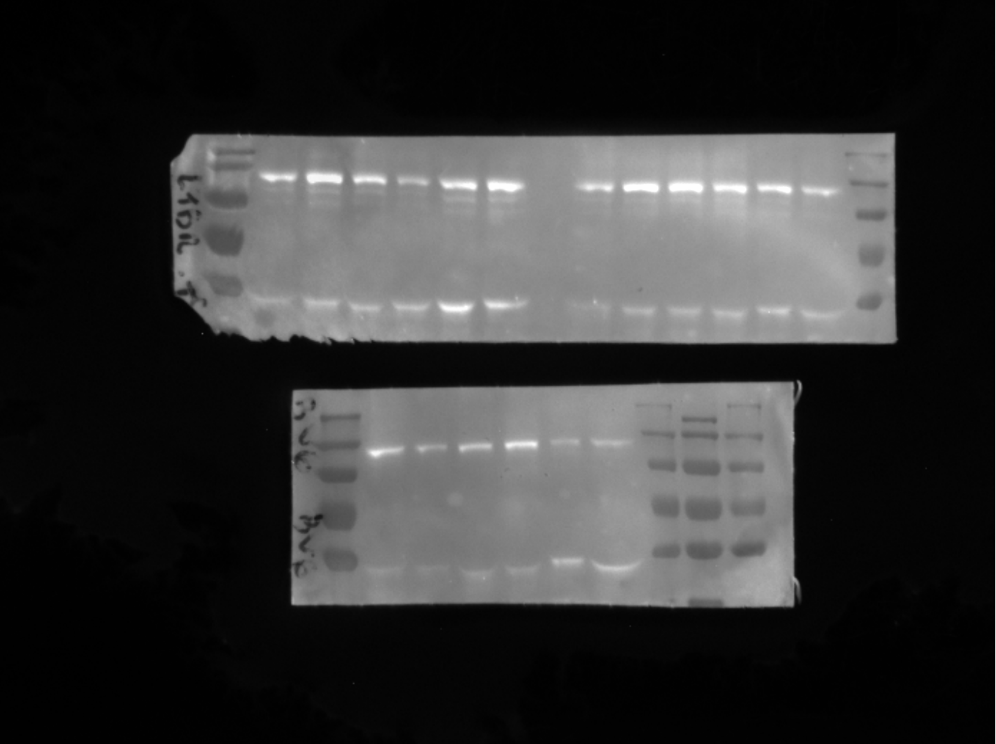


GAPDH


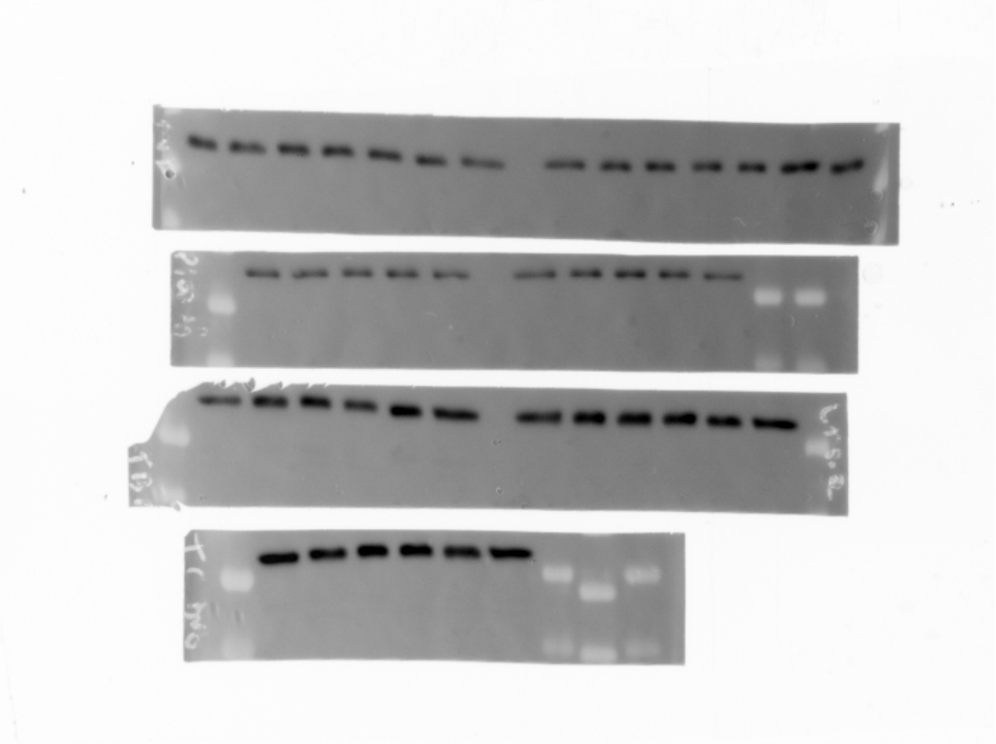


Figure supplementary 4G

NIK


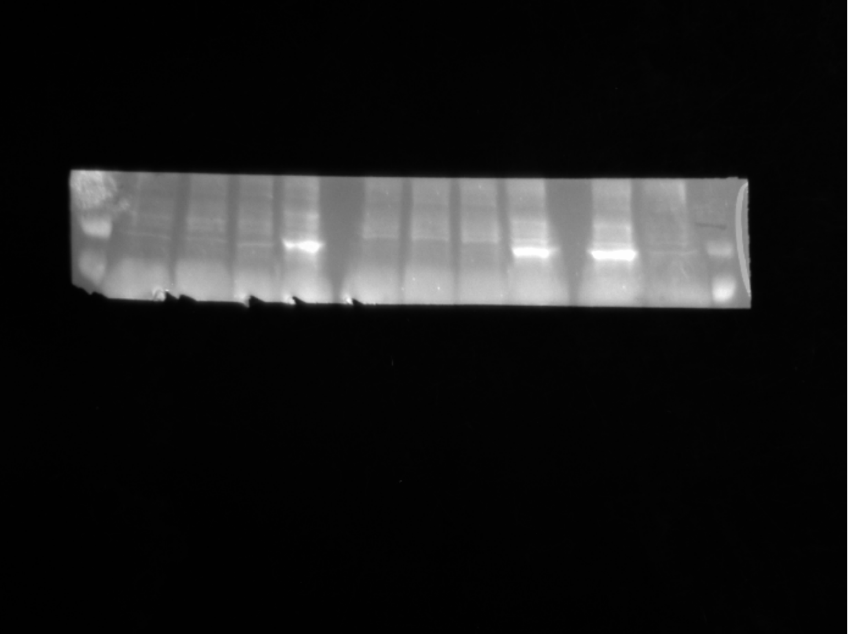


Tubulin


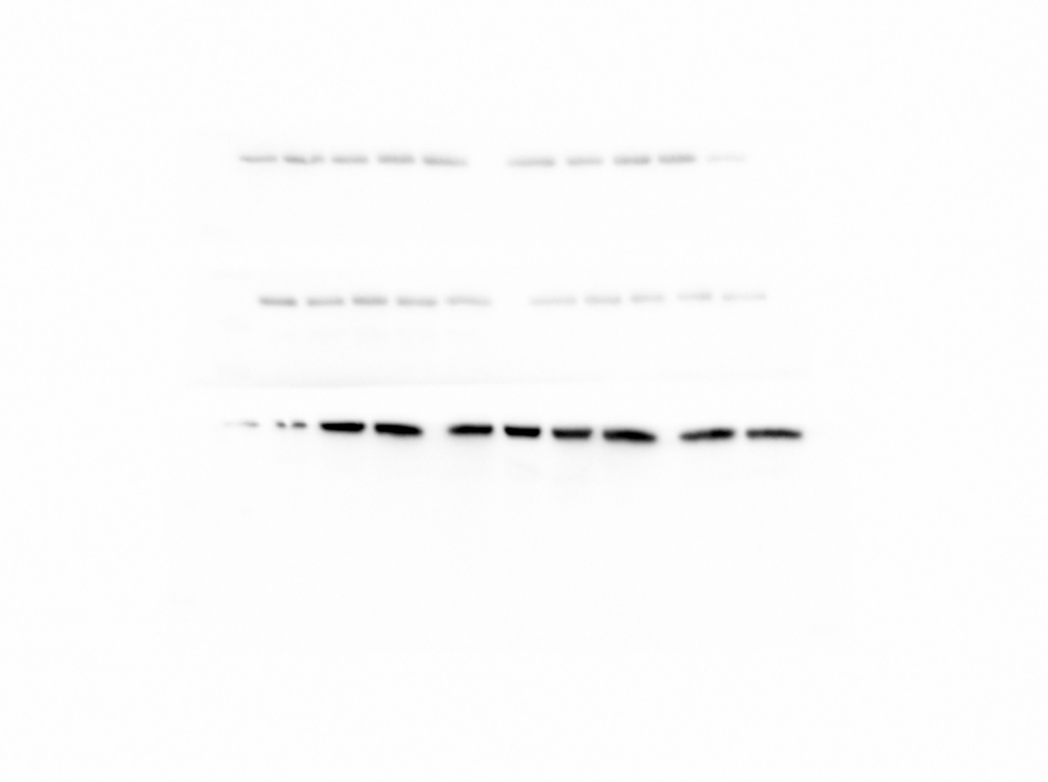


Figure supplementary 4H

P100/p52


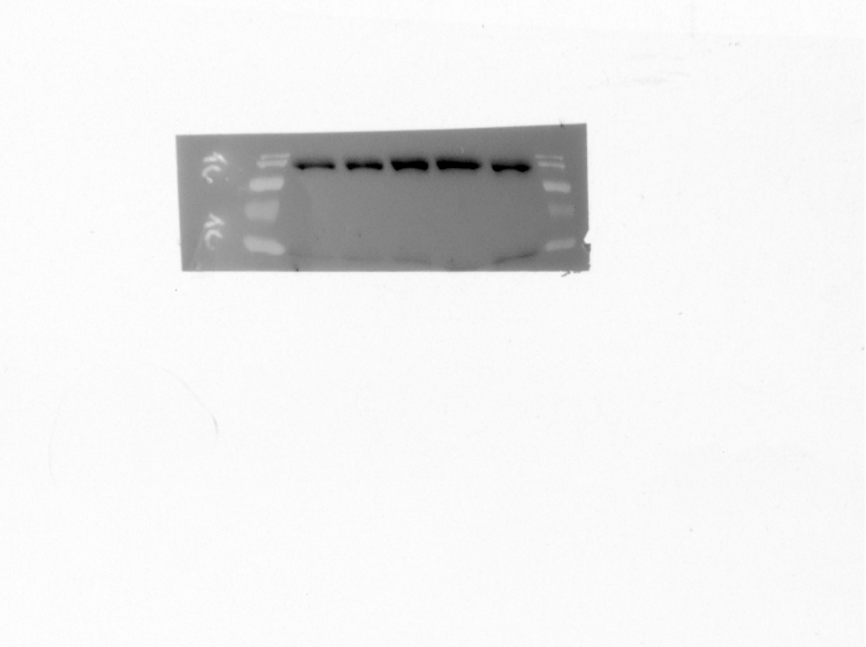


GAPDH


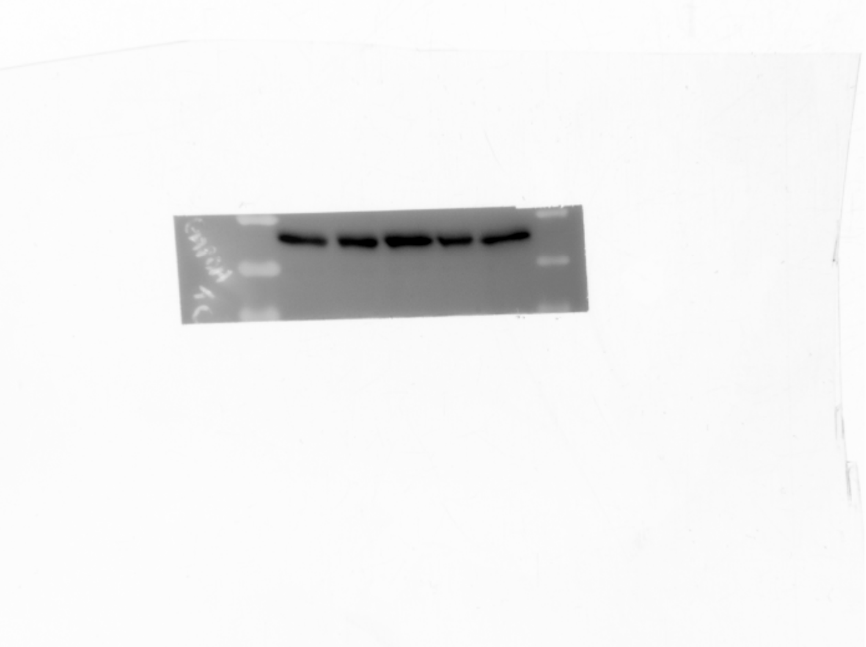

Supplement: Supplementary file 3 — Original Data File [file 41419_2022_4931_MOESM3_ESM.docx]
